# Expression and DNA methylation levels of prolyl hydroxylases *PHD1*, *PHD2*, *PHD3* and asparaginyl hydroxylase *FIH* in colorectal cancer

**DOI:** 10.1186/1471-2407-13-526

**Published:** 2013-11-06

**Authors:** Agnieszka A Rawluszko, Katarzyna E Bujnicka, Karolina Horbacka, Piotr Krokowicz, Paweł P Jagodziński

**Affiliations:** 1 Department of Biochemistry and Molecular Biology, Poznań University of Medical Sciences, Poznan, Poland; 2 Department of General and Colorectal Surgery, Poznań University of Medical Sciences, Poznan, Poland

## Abstract

**Background:**

Colorectal cancer (CRC) is one of the most common and comprehensively studied malignancies. Hypoxic conditions during formation of CRC may support the development of more aggressive cancers. Hypoxia inducible factor (HIF), a major player in cancerous tissue adaptation to hypoxia, is negatively regulated by the family of prolyl hydroxylase enzymes (PHD1, PHD2, PHD3) and asparaginyl hydroxylase, called factor inhibiting HIF (FIH).

**Methods:**

*PHD1*, *PHD2*, *PHD3* and *FIH* gene expression was evaluated using quantitative RT-PCR and western blotting in primary colonic adenocarcinoma and adjacent histopathologically unchanged colonic mucosa from patients who underwent radical surgical resection of the colon (n = 90), and the same methods were used for assessment of *PHD3* gene expression in HCT116 and DLD-1 CRC cell lines. DNA methylation levels of the CpG island in the promoter regulatory region of *PHD1*, *PHD2*, *PHD3* and *FIH* were assessed using bisulfite DNA sequencing and high resolution melting analysis (HRM) for patients and HRM analysis for CRC cell lines.

**Results:**

We found significantly lower levels of PHD1, PHD2 and PHD3 transcripts (p = 0.00026; p < 0.00001; p < 0.00001) and proteins (p = 0.004164; p = 0.0071; p < 0.00001) in primary cancerous than in histopathologically unchanged tissues. Despite this, we did not observe statistically significant differences in FIH transcript levels between cancerous and histopathologically unchanged colorectal tissue, but we found a significantly increased level of FIH protein in CRC (p = 0.0169). The reduced *PHD3* expression was correlated with significantly increased DNA methylation in the CpG island of the *PHD3* promoter regulatory region (p < 0.0001). We did not observe DNA methylation in the CpG island of the *PHD1*, *PHD2* or *FIH* promoter in cancerous and histopathologically unchanged colorectal tissue. We also showed that 5-Aza-2’-deoxycytidine induced DNA demethylation leading to increased PHD3 transcript and protein level in HCT116 cells.

**Conclusion:**

We demonstrated that reduced *PHD3* expression in cancerous tissue was accompanied by methylation of the CpG rich region located within the first exon and intron of the *PHD3* gene. The diminished expression of *PHD1* and *PHD2* and elevated level of FIH protein in cancerous tissue compared to histopathologically unchanged colonic mucosa was not associated with DNA methylation within the CpG islands of the *PHD1*, *PHD2* and *FIH* genes.

## Background

Colorectal cancer (CRC) belongs to one of the most extensively studied types of cancers due to its high mortality and severity. It is the third and second leading cause of death from malignant disease among adults in the US and Europe, respectively [1]. A decrease in oxygen concentration is widely seen during the formation of many solid tumors, including CRC. Hypoxic regions may occur due to poorly formed vasculature, shunting of blood and vascular permeability [2]. Cancer cells can adjust to this microenvironment by altering gene transcription to enhance glucose uptake and angiogenesis [2]. The various adaptive responses involve multiple mechanisms, of which the best-characterized is mediated through transcriptional gene activation by the hypoxia inducible factor (HIF) [3]. HIF is a heterodimeric transcription factor assembled from an oxygen-regulated α subunit (HIF-α) and a constitutively expressed β subunit (HIF- β) [3,4]. Under hypoxic conditions, HIF-α translocates into the nucleus, where it forms a dimer with HIF-β to form an active transcriptional complex with a number of cofactors [3,4]. The HIF complex binds to the promoter hypoxia response elements (HREs) to induce the expression of target genes that regulate the cellular adaptive response to low oxygen tension [3,4].

HIF-α is constitutively expressed in the tissue; however, it has an extremely short half-life in normoxic conditions [3]. The level of HIF-α protein is regulated in several ways. The most well known is its degradation through post-translational hydroxylation. To date, two different oxygen-dependent hydroxylation mechanisms have been identified. The first pathway is initiated by three prolyl hydroxylase domain enzymes, PHD1, PHD2 and PHD3 [3]. The second pathway involves the factor inhibiting HIF (FIH) [5]. The PHD enzymes catalyze the hydroxylation of two conserved proline residues in the oxygen dependent degradation domain of the HIF-α protein. Hydroxylated proline residues are subsequently recognized by the E3 ligase complex containing von Hippel–Lindau tumour suppressor protein (pVHL), and targeted for degradation by the 26S proteasome [3]. Similarly, FIH hydroxylates the asparagine residue within the C-terminal transactivation domain of HIF-α [5,6]. This results in the prevention of HIF-α interaction with its coactivators. Hence, under normoxic conditions, there is a dual mechanism of HIF inhibition by its degradation or inactivation by PHDs and FIH enzymes, respectively.

Recently, various studies have demonstrated inconsistent data of *FIH* and *PHD1*, *2* and *3* expression changes during CRC development [7-10]. The mechanism by which these hydroxylases might be regulated is still not well elucidated. Interestingly, *PHDs* and *FIH* genes possess a CpG island within their promoter region. Similarly to genetic mutations, hyper- or hypomethylation of gene regulatory sequences have been shown to potentially change the expression of cancer related genes in different malignancies, including CRC [11]. To date, it has been demonstrated that the promoter region of the *PHD3* gene is hypermethylated in plasma cell neoplasia, prostate, melanoma and mammary gland cancer cell lines [12,13]. The DNA methylation status of *PHD1*, *PHD2* and *FIH* has also been investigated in breast, cervical and prostate cancer cell lines, but the results are inconsistent [12,14,15]. These reports prompted us to study whether altered PHD1, PHD2, PHD3 and FIH expression levels may be correlated with the DNA methylation status of their promoter regions in primary cancerous and histopathologically unchanged colorectal tissue from the same ninety patients. We also evaluated the effect of 5-Aza-2’-deoxycytidine (5-dAzaC), an inhibitor of DNA methyltransferases (DNMTs), on the DNA methylation level of the *PHD3* gene and its effect on PHD3 transcript and protein levels in HCT116 and DLD-1 CRC cells under hypoxic and normoxic conditions.

## Methods

### Antibodies and reagents

Rabbit polyclonal (Rp) anti-PHD1 (NB100-310), -PHD2 (NB100-137), -PHD3 (NB100-139) and -FIH (NB100-428) antibodies (Ab) were provided by Novus Biologicals (Cambridge, UK). Rp anti-GAPDH Ab (FL-335) and goat anti-rabbit horseradish peroxidase (HRP)-conjugated Ab were provided by Santa Cruz Biotechnology (Santa Cruz, CA). 5-dAzaC was purchased from Sigma-Aldrich Co. (St. Louis, MO).

### Patient material

Primary colonic adenocarcinoma tissues were collected between June 2009 and July 2012 from ninety patients who underwent radical surgical resection of the colon at the Department of General and Colorectal Surgery, Poznań University of Medical Sciences, Poland (Table 1). Histopathologically unchanged colonic mucosa located at least 10–20 cm away from the cancerous lesions was obtained from the same patients. Since *ex vivo* stress may influence protein stability, one set of samples was immediately snap-frozen in liquid nitrogen and stored at -80ºC until RNA/DNA/protein isolation [16]. Another set of samples was directed for histopathological examination. Histopathological classification including stage, grade and tumour type was performed by an experienced pathologist. No patients received preoperative chemo- or radiotherapy. Written informed consent was obtained from all participating individuals. The procedures of the study were approved by the Local Ethical Committee of Poznań University of Medical Sciences.

**Table 1:** Demographic and histopathological classification including stage, grade and tumour type of patients with CRC

**Features**	**No. of patients**
Total no. of patients	90
Gender (Female/Male)	41/49
Mean (± SD) age at radical surgical resection of colon (yrs)	68.60 ± 11.45
CRC localization	
Proximal colon (cecum to transverse)	32
Distal colon (splenic flexure to sigmoid)	18
Rectum	40
Histological grade	
G1	3
G2	65
G3	22
Dukes classification	
A	8
B	35
C	47
Tumour stage	
T1	4
T2	10
T3	65
T4	11

### Cell culture

DLD-1 colon cancer cells were obtained from the American Type Culture Collection (Rockville, MD) and HCT116 cells were kindly provided by the Department of Experimental and Clinical Radiobiology, Maria Skłodowska-Curie Cancer Center, Institute of Oncology Branch, Gliwice, Poland. These cells were cultured in DMEM GibcoBRL (Grand Island, NY) containing 10% heat-inactivated fetal bovine serum (FBS) and 2 mM glutamine. To determine the effect of 5-dAzaC on DNA methylation, transcript and protein levels of selected genes, the HCT116 and DLD-1 cells were cultured for 24 hours in DMEM GibcoBRL (Grand Island, NY) supplemented with 10% FBS from Sigma-Aldrich Co. (St. Louis, MO). Cells were then cultured under normoxic or hypoxic (1% O_2_) conditions either in the absence or in the presence of 5-dAzaC at a concentration of 1.00 or 5.00 μM for 6, 24 and 48 hours. Hypoxic conditions were achieved using a MCO-18 M multigas cell culture incubator, Sanyo (Wood Dale, IL), modified to permit flushing the chamber with a humidified mixture of 5% CO_2_, 94% N_2_. These cells were used for total DNA, RNA isolation, RQ-PCR, western blotting, and HRM analysis.

### Reverse transcription and real-time quantitative polymerase chain reaction (RQ-PCR) analysis

Total RNA from primary tissues of patients with CRC and CRC cell lines was isolated according to the method of Chomczyński and Sacchi (1987) [17]. RNA samples were quantified and reverse-transcribed into cDNA. RQ-PCR was carried out in a Light Cycler®480 Real-Time PCR System, Roche Diagnostics GmbH (Mannheim, Germany) using SYBR® Green I as detection dye. The target cDNA was quantified by the relative quantification method using a calibrator for primary tissue or respective controls for HCT116 and DLD-1 cells. The calibrator was prepared as a cDNA mix from all of the patients’ samples and successive dilutions were used to create a standard curve as described in Relative Quantification Manual Roche Diagnostics GmbH, (Mannheim, Germany). For amplification, 1 μl of total (20 μl) cDNA solution was added to 9 μl of IQ™ SYBR® Green Supermix, Bio-Rad Laboratories Inc. (Hercules, CA) with primers (Additional file 1). To prevent amplification of sequences from genomic DNA contamination, primers and/or amplicons were designed at exon/exon boundaries and covered all gene splice variants (Additional file 1). The quantity of PHD1, PHD2, PHD3 and FIH transcript in each sample was standardized by the geometric mean of two internal controls. The internal control genes were *porphobilinogen deaminase* (*PBGD*) and *human mitochondrial ribosomal protein L19* (*hMRPL19*) (Additional file 1). They were selected from four candidate reference genes (*PBGD*, *hMRPL*, *peptidylprolyl isomerase A*- *PPIA*, *hypoxanthine phosphoribosyltransferase 1- HPRT*) based on the results achieved in geNorm VBA applet for Microsoft Excel (data not shown) [18,19]. The PHD1, PHD2, PHD3 and FIH transcript levels in the patients’ tissues were expressed as multiplicity of cDNA concentrations in the calibrator. In HCT116 and DLD-1 cells, transcript levels were presented as multiplicity of the respective controls.

### Western blotting analysis

Primary tissues from patients with CRC, HCT116 and DLD-1 cells were treated with lysis RIPA buffer and proteins were resuspended in sample buffer and separated on 10% Tris-glycine gel using sodium dodecyl sulfate-polyacrylamide gel electrophoresis (SDS-PAGE). Gel proteins were transferred to a nitrocellulose membrane, which was blocked with 5% milk in Tris/HCl saline/Tween buffer. Immunodetection of bands was performed with Rp anti- PHD1, -PHD2, -PHD3 and -FIH Ab, followed by incubation with goat anti-rabbit HRP-conjugated Ab. To ensure equal protein loading of the lanes, the membrane was stripped and incubated with Rp anti-GAPDH Ab (FL-335), followed by incubation with goat anti-rabbit HRP-conjugated Ab. Bands were revealed using SuperSignal West Femto Chemiluminescent Substrate, Thermo Fisher Scientific (Rockford, IL) and Biospectrum® Imaging System 500, UVP Ltd. (Upland, CA). The amounts of analyzed proteins were presented as the protein-to-GAPDH band optical density ratio. For HCT116 and DLD-1 cells cultured in the absence of 5-dAzaC, the ratio of PHD3 to GAPDH was assumed to be 1.

### DNA isolation and bisulfite modification

Genomic DNA was isolated using DNA Mammalian Genomic Purification Kit purchased from Sigma-Aldrich Co. (St. Louis, MO). 500 ng of genomic DNA was subjected to bisulfite conversion of cytosine to uracil according to the EZ DNA Methylation Kit™ procedure from Zymo Research Corporation (Orange, CA). The position of CpG islands and binding sites of transcription factors located in the regulatory region of the promoter was determined by online programs [20-22].

### DNA methylation evaluation by bisulfite sequencing

DNA fragments containing CpG dinucleotides located in the promoter region of the *PHD1*, *PHD2*, *PHD3* and *FIH* genes were amplified from the bisulfite-modified DNA by the primer pairs (Additional file 1, Additional file 2) complementary to the bisulfite-DNA modified sequence. PCR amplification was performed by FastStart Taq DNA Polymerase from Roche Diagnostic GmbH (Mannheim, Germany). The PCR products were purified using Agarose Gel DNA Extraction Kit, Roche Diagnostic GmbH (Mannheim, Germany) with subsequent cloning into pGEM-T Easy Vector System I, Promega (Madison, WI) and transformation into TOPO10 *E. coli* strain cells. Plasmid DNA isolated from five positive bacterial clones was used for commercial sequencing of the cloned fragment of DNA. The results of bisulfite sequencing were assessed and presented using BiQ analyzer software and Bisulfite sequencing Data Presentation and Compilation (BDPC) web server, respectively [23,24].

### DNA methylation assessment by high resolution melting (HRM) analysis

Methylation levels of DNA fragments located within the CpG island of the *PHD1*, *PHD2*, *PHD3* and *FIH* genes (Additional file 2) were determined by Real Time PCR amplification of bisulfite treated DNA followed by HRM profile analysis by Light Cycler®480 Real-Time PCR System, Roche Diagnostics GmbH (Mannheim, Germany). For PCR amplification, 1 μl of the bisulfite treated DNA from patients, HCT116, DLD-1 cells, or standards, and primers (Additional file 1, Additional file 2) was added to 19 μl of 5 X Hot FIREPol EvaGreen HRM Mix, Solis BioDyne Co. (Tartu, Estonia). Standardized solutions of DNA methylation percentage were prepared by mixing methylated and non-methylated bisulfite treated DNA from Human Methylated/Non-methylated DNA Set, Zymo Research Corp. (Orange, CA) in different ratios. To determine the percentage of methylation, the HRM profiles of patient DNA PCR products were compared with HRM profiles of standard DNA PCR product [25,26]. HRM methylation analysis was performed using Light Cycler®480 Gene Scanning software, Roche Diagnostics GmbH (Mannheim, Germany). Each PCR amplification and HRM profile analysis was performed in triplicate. Using HRM analysis we were able to detect heterogenous methylation with equal sensitivity (Additional file 3). The methylation for each patient was presented as a percentage of methylation in amplified fragments located in the CpG island of *PHD1, PHD2, PHD3* and *FIH*. Since low levels of methylation may not demonstrate significant biological effect and we are not able to quantify all CpG dinucleotides within the analyzed CpG island, the percentage results were divided into three groups: 0–1% methylation, 1–10% methylation and 10–100% methylation for statistical analysis [27-30].

### Statistical analysis

The normality of the observed patient data distribution was assessed by Shapiro-Wilk test, and unpaired, two-tailed *t*-test or U Mann–Whitney test was used to compare the mean values. The chi-square test was used to examine significance in DNA methylation. To evaluate the association between different ranges of DNA methylation (0–1% methylation, 1–10% methylation and 10–100% methylation) and the ratio of cancerous tissue PHD3 mRNA level to histopathologically unchanged PHD3 mRNA level, the non-parametric Kruskal-Wallis test was employed. Data groups for cell lines were assessed by ANOVA to evaluate if there was significance (P < 0.05) between the groups. For all experimental groups, which fulfilled the initial criterion, individual comparisons were performed by post hoc Tukey test with the assumption of two-tailed distribution. Statistically significant results were indicated by p < 0.05. Statistical analysis was performed with STATISTICA 6.0 software.

## Results

### PHD1, PHD2, PHD3 and FIH transcript and protein levels in primary cancerous and histopathologically unchanged tissues from patients with CRC

**Figure 1:**
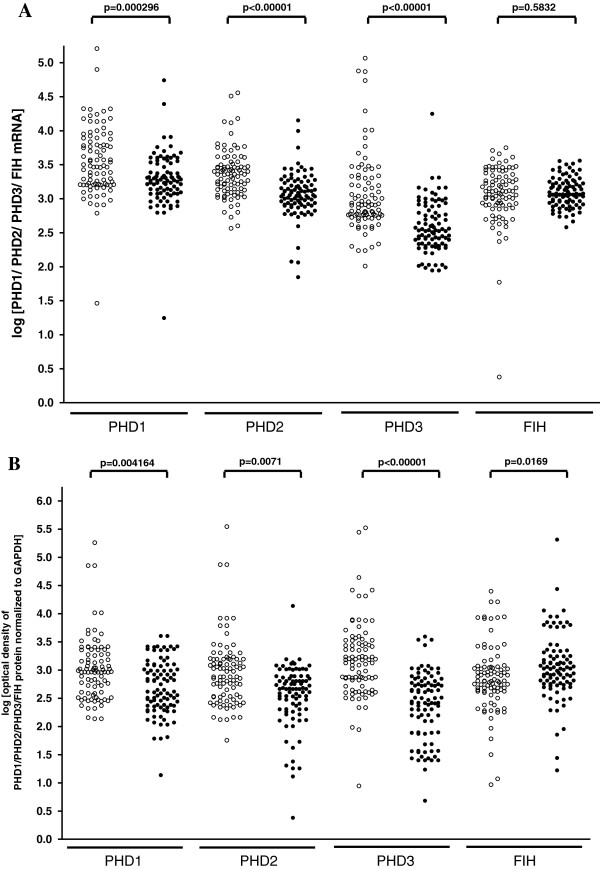
**PHD1, PHD2, PHD3 and FIH transcript and protein levels in primary cancerous and histopathologically unchanged tissues from patients with CRC. A.** The cancerous (●) and histopathologically unchanged tissues (○) from ninety patients with CRC were used for RNA and protein isolation. Total RNA was reverse-transcribed, and cDNAs were investigated by RQ-PCR relative quantification analysis. The PHD1, PHD2, PHD3 and FIH mRNA levels were corrected by the geometric mean of PBGD and hMRPL19 cDNA levels. The amounts of PHD1, PHD2, PHD3 and FIH mRNA were expressed as the decimal logarithm of multiples of these cDNA copies in the calibrator. **B.** Proteins were separated by 10% SDS-PAGE, and transferred to a membrane that was then immunoblotted with Rp anti- PHD1, - PHD2, - PHD3 and - FIH Ab and incubated with goat anti-rabbit HRP-conjugated Ab. The membrane was then stripped and blotted with Rp anti-GAPDH Ab, followed by incubation with goat anti-rabbit HRP-conjugated Ab. The amount of western blot-detected PHD1, PHD2, PHD3 and FIH proteins was presented as the decimal logarithm of PHD1, PHD2, PHD3 and FIH to GAPDH band optical density ratio. The p value was evaluated by unpaired, two-tailed t-test or U-Mann-Whitney test.

**Figure 2:**
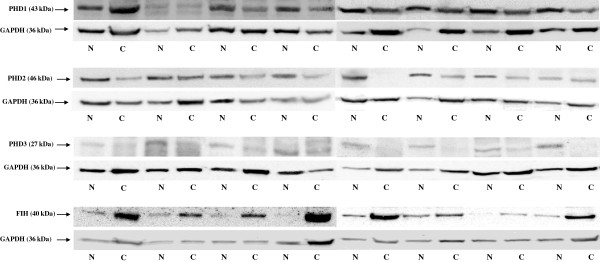
**Representative picture of western blot in histopathologically unchanged tissue (N) and primary cancerous tissue (C) from patients with CRC.** Immunodetection of bands was performed with Rp anti- PHD1, - PHD2, - PHD3 and - FIH Ab, followed by incubation with goat anti-rabbit HRP-conjugated Ab. The membrane was stripped and incubated with Rp anti-GAPDH Ab, followed by incubation with goat anti-rabbit HRP-conjugated Ab. Bands were revealed using SuperSignal West Femto Chemiluminescent Substrate, Thermo Fisher Scientific (Rockford, IL) and Biospectrum® Imaging System 500, UVP Ltd. (Upland, CA).

To compare PHD1, PHD2, PHD3, and FIH transcript and protein levels in cancerous and histopathologically unchanged tissues from ninety patients with CRC we used RQ-PCR and western blotting, respectively. We found significantly lower levels of PHD1, PHD2 and PHD3 transcript (p = 0.00026; p < 0.00001; p < 0.00001) and protein (p = 0.004164; p = 0.0071; p < 0.00001) in primary cancerous than in histopathologically unchanged tissues in ninety patients with CRC (Figure 1A, B; Figure 2). Moreover, we observed significantly lower levels of PHD1, PHD2, PHD3 transcript and protein in cancerous tissue in different age groups, among the genders, CRC localization, G2 and G3 histologic grade, levels of Dukes scale [31], and tumour stage (Additional file 4). There was no significant difference in the levels of FIH transcript between primary cancerous and histopathologically unchanged tissues in ninety patients with CRC (p = 0.583) (Figure 1A). However, we observed a statistically higher level of FIH protein in primary cancerous than in histopathologically unchanged tissue (p = 0.0169) (Figure 1B, Figure 2). We also found a significantly higher level of FIH protein in cancerous tissue in the male patient group (p = 0.0210), and in patients aged above 60 (p = 0.0257), with CRC localized in the rectum (p = 0.031) and G2 histologic grade (p = 0.0226) (Additional file 4).

### DNA methylation levels in primary cancerous and histopathologically unchanged tissues from patients with CRC

**Figure 3:**
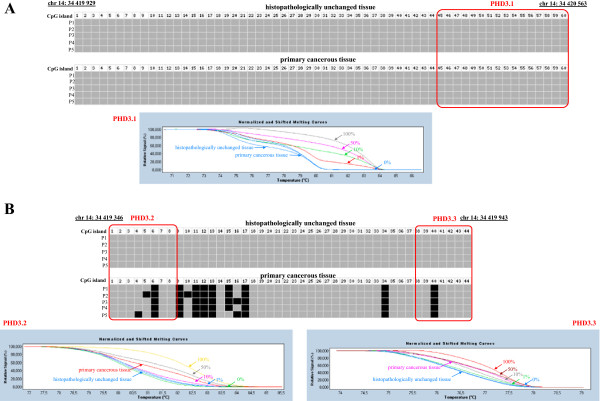
**DNA methylation assessment of *PHD3 *gene regulatory region by bisulfite sequencing and HRM analysis in primary tissue samples from patients with CRC.** Primary cancerous and histopathologically unchanged tissues from the same patients with CRC (P1-P5) were used for genomic DNA isolation followed by bisulfite conversion of cytosine to uracil. The *PHD3* regions containing 60 CpG dinucleotides (chr14: 34 419 929-34 420 563) (Top panel **A**) and 44 CpG dinucleotides (ch14: 34 419 346-34 419 943) (Top panel **B**) were then amplified by a pair of primers complementary to the bisulfite-DNA modified sequence (Additional file 1, Additional file 2). The PCR products were purified with subsequent cloning into a plasmid vector. Plasmid DNA isolated from five positive bacterial clones was used for commercial sequencing. The results of bisulfite sequencing were assessed and presented using BiQ analyzer software and BDPC web server [23,24]. Black and grey boxes represent methylated and unmethylated CpG dinucleotide, respectively. Red rectangles correspond to regions amplified in HRM analysis by specific primers PHD3.1 (chr14: 34 419 922-34 420 080), PHD3.2 (chr14: 34 419 795- 34 419 935) and PHD3.3 (chr14: 34 419 400-34 419 538) (Additional file 1, Additional file 2). Bottom panels A and B represent HRM profiles of standard and example of patient DNA (patient P2 from bisulfite sequencing) PCR product. Methylation percentage of three DNA fragments within the *PHD3* CpG island was determined by Real Time PCR amplification of bisulfite treated standard and patient DNA, followed by comparison of their HRM profiles. DNA standards were prepared by mixing different ratios of methylated and non-methylated bisulfite treated DNA. HRM methylation analysis was performed using Light Cycler®480 Gene Scanning software, Roche Diagnostics GmbH (Mannheim, Germany). Each PCR amplification and HRM profile analysis was performed in triplicate.

**Figure 4:**
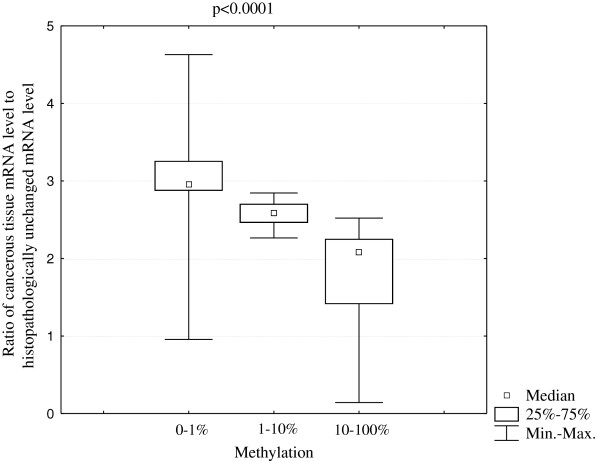
**Ratio of cancerous PHD3 mRNA level to histopathologically unchanged tissue PHD3 mRNA level in three ranges of *PHD3 *methylation status: 0–1%; 1–10% and 10–100%.** Methylation percentage of three DNA fragments within the *PHD3* CpG island (Additional file 1, Additional file 2) was determined by Real Time PCR amplification of bisulfite treated standard and patient DNA, followed by comparison of their HRM profiles. The methylation for each patient was calculated as an average percentage of methylation in amplified fragments located in the CpG island of *PHD3.* The samples were divided into three groups for statistical analysis: 0–1% methylation, 1–10% methylation and 10–100% methylation (Table 2) [28-30]. To evaluate the statistically significant difference in the ratio of cancerous PHD3 mRNA level to histopathologically unchanged tissue PHD3 mRNA level between the three DNA methylation ranges (0–1% methylation, 1–10% methylation and 10–100% methylation), the non-parametric Kruskal-Wallis test was employed.

To compare DNA methylation levels in the promoter region of the *PHD1*, *PHD2*, *PHD3*, and *FIH* genes between DNA samples from cancerous and histopathologically unchanged tissues, we performed sodium bisulfite DNA sequencing and HRM analysis (Additional file 1, Additional file 2). Bisulfite sequencing was used for preliminary evaluation of DNA methylation in large regions of selected CpG islands in randomly selected patients. We detected a similar pattern of DNA methylation within all individual clones of each patient. The DNA methylation level evaluation for *PHD3* revealed significant differences between cancerous and histopathologically unchanged tissue in region chr14: 34 419 346–34 419 943 (Figure 3B, Additional file 1, Additional file 2). However, we observed no changes of DNA methylation within the promoter of *PHD3* in region chr14: 34 419 929–34 420 563 (Figure 3A, Additional file 1, Additional file 2). Moreover, we did not detect DNA methylation in the regulatory region of the *PHD1*, *PHD2* and *FIH* genes in cancerous and histopathologically unchanged tissue in selected patients with CRC (Additional file 1, Additional file 2, Additional file 5). To extend DNA methylation studies and to confirm bisulfite sequencing data for all analyzed genes, we employed HRM analysis of PCR amplified bisufite treated DNA for patients. Depending on the length of the CpG island and the amplification possibilities of bisulfite treated DNA, one to three primer pairs was used in HRM analysis (Additional file 1, Additional file 2). In keeping with the bisulfite sequencing data, we observed no DNA methylation within the promoter region of the *PHD1*, *PHD2* and *FIH* genes in cancerous and histopathologically unchanged tissue from ninety patients with CRC (Additional file 1, Additional file 2, Additional file 6). We also detected no DNA methylation for *PHD3* in region chr14: 34 419 922–34 420 080 in cancerous and histopathologically unchanged tissue using HRM analysis (Figure 3A, Additional file 1, Additional file 2). However, HRM evaluation showed a significant increase in the average DNA methylation level in cancerous compared to histopathologically unchanged tissue from ninety patients with CRC in the CpG island of the *PHD3* gene in regions chr14: 34 419 795–34 419 935 and chr14: 34 419 400–34 419 538 (p < 0.00001) (Figure 3B; Table 2, Additional file 1, Additional file 2). HRM results were compared with those obtained in bisulfite sequencing for all analyzed genes in reconstituted samples. A similar pattern of DNA methylation was observed between these two methods (Figure 3). Moreover, we observed that an increase in the average DNA methylation level of *PHD3* in regions chr14: 34 419 795–34 419 935 and chr14: 34 419 400–34 419 538 correlated to a decrease in the ratio of cancerous-to-histopathologically-unchanged tissue PHD3 mRNA level (p < 0.0001) (Figure 4).

**Table 2:** **Methylation level of the regulatory region of the *PHD3 *** **gene in primary cancerous tissue and histopathologically unchanged tissue sample from patients with CRC**

	**Total no. of patients**	**< 1% methylation**	**1–10% methylation**	**10–100% methylation**	**p**^**a**^
**Histopathologically unchanged tissue**	90	63	27	0	p < 0.00001
**Primary cancerous tissue**		37	31	22	

The primary cancerous and histopathologically unchanged tissue samples from the same patients were used for genomic DNA isolation, followed by bisulfite conversion of cytosine to uracil. The DNA fragments of the CpG island were then amplified by three pairs of primers complementary to the bisulfite-DNA modified sequence (Additional file 1, Additional file 2). To determine the percentage of methylation, the HRM profiles of the patients’ DNA PCR products were compared to HRM profiles of the prepared standard PCR products (Figure 3). DNA methylation of the *PHD3* regulatory region for each patient was calculated as mean of the percentage of methylation in two DNA amplified fragments where the differences between primary cancerous and histopathologically unchanged tissue where observed. ^a^ Chi square test.

### DNA methylation level of the *PHD1*, *PHD2* and *FIH* genes in HCT116 and DLD-1 CRC cells

To assess DNA methylation levels in the promoter region of the *PHD1*, *PHD2*, and *FIH* genes in DLD-1 and HCT116 cells, we performed HRM analysis (Additional file 1, Additional file 2). We observed no DNA methylation of the promoter region of *PHD1*, *PHD2* and *FIH* gene in the analyzed regions using HRM analysis under hypoxic and normoxic conditions (Additional file 6).

### The hypermethylated *PHD3* gene in HCT116 is not induced upon hypoxia conditions

**Figure 5:**
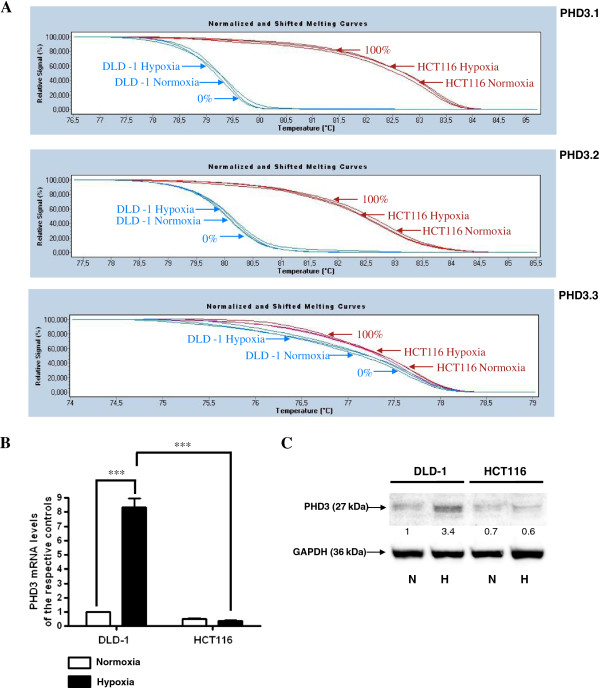
**DNA methylation and expression level of the *PHD3 *gene in HCT116 and DLD-1 CRC cells. A.** HCT116 and DLD-1 cells were cultured under normoxic or hypoxic (1% O_2_) conditions for 48 hrs. Cells were then used for DNA isolation followed by bisulfite modification. Methylation percentage of three DNA fragments within the *PHD3* CpG island (Additional file 1, Additional file 2) in HCT116 and DLD-1 cells under hypoxic and normoxic conditions was determined by Real Time PCR amplification of bisulfite treated standard and cell line DNA, followed by comparison of their HRM profiles. **B.** Cells were cultured in DMEM either in hypoxic (1%O_2_) or normoxic conditions for 48 hrs. After incubation, the cells were used for total RNA isolation and reverse transcription. The PHD3 cDNA levels were determined by RQ-PCR relative quantification analysis. RQ-PCR results were standardized by the geometric mean of PBGD and hMRPL19 cDNA levels. PHD3 cDNA levels are expressed as a multiplicity of these cDNA copies in the cell line’s calibrator. **C.** Cells were cultured in DMEM either in hypoxic (1%O_2_) (**H**) or normoxic (**N**) conditions for 48 hrs. Cells were then used for protein isolation. Proteins were separated by 10% SDS-PAGE, and transferred to a membrane that was then immunoblotted with Rp anti - PHD3 Ab and incubated with goat anti-rabbit HRP-conjugated Ab. The membrane was then stripped and reblotted with Rp anti-GAPDH Ab, followed by incubation with goat anti-rabbit HRP-conjugated Ab. The band densitometry readings were normalized to GAPDH loading control. The ratio of PHD3 to GAPDH for DLD-1 in normoxic conditions was assumed to be 1.

To evaluate the association between DNA methylation of the *PHD3* gene and its expression in HCT116 and DLD-1 CRC cell lines we performed HRM analysis, RQ-PCR, and western blotting. We observed a high level of DNA methylation in HCT116 and no DNA methylation in DLD-1 cells in the chr14: 34 419 922–34 420 080, chr14: 34 419 795–34 419 935 and chr14: 34 419 400–34 419 538 regions of *PHD3* gene CpG island using HRM analysis in both hypoxic and normoxic conditions (Figure 5A). We detected a lower level of PHD3 transcript and protein in HCT116 cells compared to DLD-1 cells in both hypoxic and normoxic conditions (Figure 5B, C). However, statistical significance in these differences occurred only under hypoxic conditions (Figure 5B, C). Moreover, we observed a statistically significant induction of *PHD3* transcript and protein level upon hypoxia in DLD-1 cells, with no changes in HCT116 cells under the same conditions (Figure 5B, C).

### 5-dAzaC induced DNA demethylation of *PHD3* promoter region, PHD3 transcript and protein contents in HCT116 cells, and did not affect *PHD3* DNA methylation or expression levels in DLD-1 cells under hypoxic and normoxic conditions

**Figure 6:**
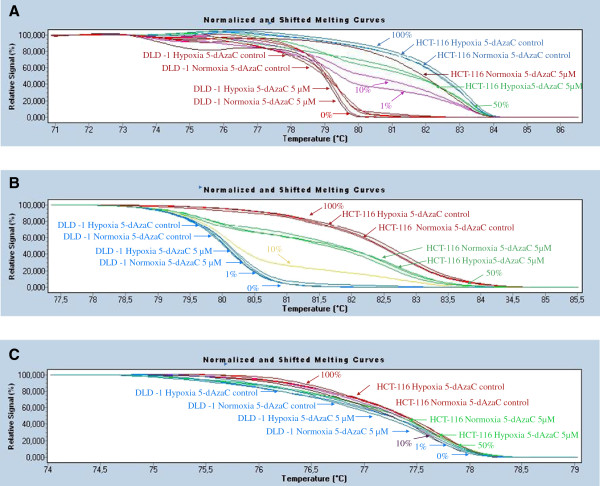
**Effect of 5-dAzaC on *PHD3 *gene DNA methylation in HCT116 and DLD-1 CRC cells.** HCT116 and DLD-1 cells were cultured under normoxic or hypoxic (1% O_2_) conditions either in the absence or in the presence of 5-dAzaC at a concentration of 5.00 μM for 48 hrs. Cells were then used for DNA isolation followed by bisulfite modification. Methylation percentage of three DNA fragments within the *PHD3* CpG island: **A** (chr14: 34 419 922–34 420 080), **B** (chr14: 34 419 795–34 419 935) and **C** (chr14: 34 419 400–34 419 538) (Additional file 1, Additional file 2) in HCT116 and DLD-1 cells under hypoxic and normoxic conditions was determined by Real Time PCR amplification of bisulfite treated standard and cell line DNA, followed by comparison of their HRM profiles.

**Figure 7:**
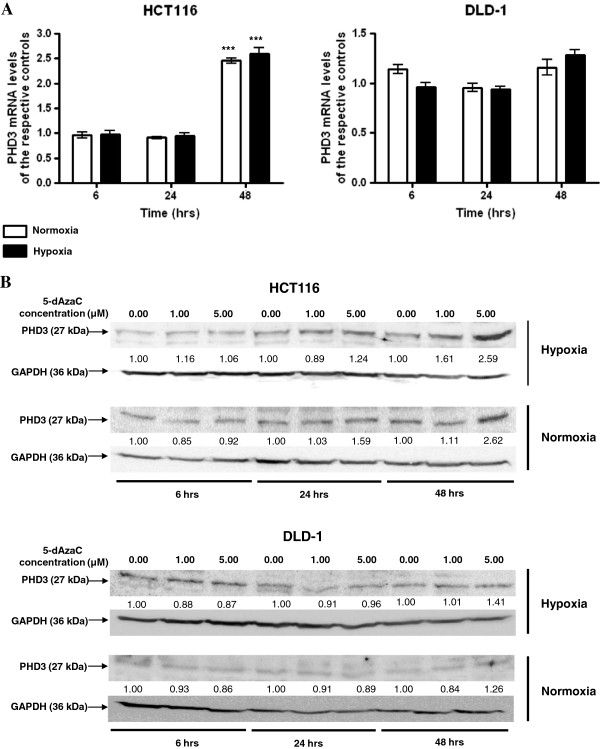
**5-dAzaC effect on PHD3 transcript (A) and protein (B) levels in HCT116 and DLD-1 CRC cells.** HCT116 and DLD-1 cells were cultured in DMEM for 6, 24 and 48 h either in the absence or in the presence of 5-dAzaC at a concentration of 1.00 or 5.00 μM under hypoxic or normoxic conditions. After incubation the cells were used for total RNA isolation and protein isolation. Total RNA was reverse-transcribed, and PHD3 cDNA levels were determined by RQ-PCR relative quantification analysis. RQ-PCR results were standardized by the geometric mean of PBGD and hMRPL19 cDNA levels. PHD3 cDNA levels are expressed as a multiplicity of the respective controls. Each sample was determined in triplicate and results are presented as the mean ± SE from three experiments *P < 0.05. The cell protein was separated by 10% SDS-PAGE, and transferred to a membrane that was then immunoblotted with Rp anti - PHD3 Ab and incubated with goat anti-rabbit HRP-conjugated Ab. The membrane was then stripped and reblotted with Rp anti-GAPDH Ab, followed by incubation with goat anti-rabbit HRP-conjugated Ab. The band densitometry readings were normalized to GAPDH loading control. The ratio PHD3 to GAPDH for control was assumed to be 1.

In order to assess the effect of 5-dAzaC on DNA methylation and *PHD3* gene expression levels we used HRM analysis, RQ-PCR, and western blotting. We observed no effect of 5-dAzaC treatment on the DNA methylation status in the analyzed regions of the *PHD3* promoter region in DLD-1 cells upon hypoxic and normoxic conditions (Figure 6A, B, C). On the contrary, using HRM analysis we noticed significant DNA demethylation in chr14: 34 419 922–34 420 080, chr14: 34 419 795–34 419 935 and chr14: 34 419 400–34 419 538 regions of the CpG island of the *PHD3* gene in HCT116 cells cultured for 48 hrs in the presence of 5.00 μM 5-dAzaC in both hypoxic and normoxic conditions (Figure 6A, B, C). The changes in DNA methylation level were accompanied by 5-dAzaC induced expression of *PHD3* in HCT116 cells. We observed that 5-dAzaC resulted in a progressive increase in PHD3 transcript levels in HCT116 cells and no significant changes for DLD-1 cells (Figure 7A). For HCT116 we found approximately a 2.45- and 2.59-fold significant increase in PHD3 transcript levels at 48 hrs of incubation under normoxic and hypoxic conditions, respectively (Figure 7A). Alterations in PHD3 transcript levels in HCT116 cells were associated with increased PHD3 protein levels in both hypoxic and normoxic conditions (Figure 7B). Densitometric analysis of western blotting bands indicated an approximately 2.59- and 2.62- fold increase in PHD3 protein level in HCT116 cells incubated with 5.00 μM 5-dAzaC for 48 hrs as compared to the respective controls under hypoxic and normoxic conditions, respectively. Incubation of DLD-1 cells with 5-dAzaC at various concentrations for different time periods did not significantly increase PHD3 protein contents under either hypoxic or normoxic conditions (Figure 7B).

## Discussion

The maintenance of oxygen homeostasis is a crucial physiological process [32]. Hypoxia is a common feature of human cancers, associated with higher aggressiveness and resistance to chemotherapy [33,34]. The cellular environment response to hypoxia is mediated by HIF, a heterodimeric transcriptional complex, which is post-translationally regulated by prolyl and asparaginyl hydroxylases. The first group, PHD enzymes, govern the ubiquitin- mediated mechanism of HIF-α degradation under normoxic conditions [3]. Since hydroxylation reactions require the presence of oxygen, dioxygenases are unable to function in hypoxic conditions. Thus, the HIF is not directed to proteasomal degradation and may exert its effect on genes during CRC progression. However, oxygen concentration is not the only cause of altered PHD function during tumorigenesis. The contribution of PHD to cell behaviour depends on several conditions, including the relative abundance of PHD proteins in a specific tissue [3]. Since changes in the amount of available PHD enzymes will alter the rate of hydroxylation of HIF-α, research has focused on the regulation of the quantity of PHDs. Aberrant expression of PHDs has been observed in a variety of human cancers. Detailed analysis of the expression patterns of all three PHD isoforms has shown differences depending on cancer cell type [7,35-37].

PHD1 protein levels were elevated in non-small cell type lung cancer (NSCLC) and pancreatic endocrine tumors [36,38]. Moreover, PHD1 inactivation resulted in lower levels of Cyclin D1 and impaired breast tumour formation [39]. However, it has been demonstrated that colon cancer cells expressing the *PHD1* gene cause inhibition of tumour growth and angiogenesis under hypoxic conditions when injected into nude mice [40].

PHD2 was also previously studied in various cancer types. Low levels of *PHD2* expression were correlated with poor survival rate in CRC and breast cancer [9,41]. Nonetheless, studies in head and neck squamous cell carcinoma (HNSCC) demonstrated an association between increased levels of nuclear PHD2 protein with strongly proliferative and malignant tumour phenotypes [42,43]. Furthermore, immunohistochemical studies have found that high *PHD2* and *PHD3* expression was significantly associated with worse 5-year survival rate in pancreaticobiliary cancer [44]. PHD3 transcript levels were shown to be increased in HNSCC, and *in vitro* analysis revealed inhibition of cell cycle progression in cancerous cells in the absence of PHD3 activity [45]. Elevated levels of PHD3 protein in cancerous tissue were also observed in immunohistochemical studies of NSCLC and gastric cancer [38,46]. However, PHD3 catalytic activity has demonstrated an ability to induce apoptosis independent of HIF in different cancer cell lines [12,47-49]. Additionally, Peurala et al. presented that increased PHD3 expression and was associated with an increased survival rate in breast cancer patients [41].

Discrepancies between *PHD1*, *PHD2* and *PHD3* experimental results might be partly explained by the demonstration that HIF and PHDs can form a feedback loop that limits hypoxic signaling in reoxygenated cells [50]. Moreover, these discrepancies imply a dual function of PHDs in the control of tumour progression that depends on cell type, other PHDs- interacting factors, and function independent of HIF. The HIF- independent functions of PHDs include PHD3 directed inhibition of phosphorylation of the inhibitor of κβ kinase β and activation of NF κβ in CRC, neuronal apoptosis and myoblast differentiation by PHD3, or control of myocardial development by PHD2 [8,51,52].

We found a statistically significant decrease in the expression levels of the *PHD1*, *PHD2* and *PHD3* genes in cancerous tissue compared to histopathologically unchanged colorectal tissue. Although we did not observe statistically significant differences in FIH transcript levels between cancerous and histopathologically unchanged colorectal tissue, we found a significant increase of FIH protein in CRC. FIH, in addition to PHDs, hydroxylates HIF-α at a conserved asparagine residue. Through this modification, FIH prevents interaction of HIF with its transcriptional coactivators [5]. More importantly, FIH is able to suppress HIF activity under severe hypoxic conditions, where PHDs are inactive [53,54]. Additionally, FIH exerts HIF-independent functions by interaction with pVHL, histone deacetylases, p105, Notch 1, and SOCS box proteins [55-58]. FIH is widely expressed; however, its expression varies between tissue and cancer types [7]. To date, FIH protein overexpression was correlated with a more malignant phenotype and poor prognosis in pancreatic endocrine tumours and NSCLC, respectively [36,38]. Moreover, it has been established that *FIH* gene silencing reduced colon cancer cell proliferation *in vitro* and *in vivo* through the p53-p21 mediated pathway [54]. We observed a significant increase in the protein level of FIH in CRC tissue. This observation may result from E3 ligase activity of a member of seven in absentia homolog 1 (SIAH-1). Therefore, SIAH-1 facilitates ubiquitination and proteasomal degradation of FIH [59,60]. To date, SIAH-1 has been found to be widely distributed in human cell lines and tissues, including CRC, with a decreased expression in breast and hepatocellular cancer [61-63].

Since the prolyl and asparaginyl hydroxylases have so wide and profound an effect on tumorigenesis, studies on the regulation of the expression of these enzymes may help in our understanding of CRC progression. There are many factors involved in the development and occurrence of CRC, and they are classified as genetic, epigenetic and environmental [64-67]. One of the epigenetic mechanisms, namely altered DNA methylation in a gene’s regulatory region, is observed from the early stages of lesions in aberrant crypt foci and hyperplastic polyps [68,69]. It has been shown that DNA hypermethylation of *MLH1*, *MGMT*, *CDKN2A* and many others is associated with CRC progression [67,70-72]. Our study is the first to investigate the methylation status of the promoter regulatory regions of *PHD1*, *PHD2*, *PHD3* and *FIH* in primary cancerous tissue from patients with CRC, and HCT116, DLD-1 CRC cell lines. We did not observe DNA methylation within the CpG island of the *PHD1*, *PHD2* and *FIH* gene promoter in either patients or cell lines. To date, the DNA methylation status of the promoter region of *PHD1*, *PHD2*, *PHD3* and *FIH* was studied in a few cancers, including breast, prostate, cervical, melanoma, kidney and plasma cell neoplasia [12-15]. In cervical cancer cell lines the hypomethylation of the CpG island of the *PHD2* gene promoter was associated with an increase in *PHD2* expression [15]. Similarly to our results, no DNA methylation within the CpG island of *PHD1*, *PHD2* and *FIH* has been observed in breast and plasma cell neoplasia [13,14]. There was also no DNA methylation in the promoter region of the *PHD3* gene in clinical samples from breast and prostate cancer [12,14]. We also observed no DNA methylation within the *PHD3* gene using bisulfite sequencing in region chr14: 34 419 929–34 420 563 and HRM analysis in region chr14: 34 419 922–34 420 080 in a group of patients, which is consistent with the results of Huang et al. and Place et al. [12,14]. However, we found a significantly higher level of DNA methylation within the first exon and intron of the *PHD3* gene (chr14: 34 419 346–34 419 943 using bisulfite sequencing; chr14: 34 419 795–34 419 935 and chr14: 34 419 400–34 419 538 using HRM analysis) in cancerous tissue compared to histopathologically unchanged tissue. To the best of our knowledge, DNA methylation within the chr14: 34 419 346–34 419 943, chr14: 34 419 795–34 419 935 and chr14: 34 419 400–34 419 538 regions was not previously analyzed in other studies. Data from Encyclopedia of DNA elements project showed that these regions are DNase I hypersensitive and able to bind various transcription factors, which suggests a promoter or enhancer activity [22]. Moreover, Pescador et al. (2005) identified a functional HRE within the first intron of the *PHD3* gene and proposed a model of HIF-mediated hypoxic induction of PHD3 [73].

Since hypoxic conditions may induce global DNA hypomethylation in cancer cells, we investigated DNA methylation and expression levels of the *PHD3* gene in HCT116 and DLD-1 cells under hypoxic and normoxic conditions [74]. We reported a high level of DNA methylation and no transcript and protein level changes under hypoxic and normoxic conditions in HCT116 cells. In the *PHD3* gene promoter region in DLD-1 cells, we did not detect DNA methylation under either experimental condition, but we observed a significant induction of PHD3 transcript and protein level upon hypoxia. The *PHD3* gene possesses in its promoter region a putative HRE and can be induced by HIF transcription factor complex under hypoxic conditions [35]. A lack of increase in *PHD3* expression in HCT116 cells might be the result of DNA methylation of its promoter region in hypoxic conditions. To date, a decreased expression of PHD3 mRNA was correlated with high CpG island methylation status in plasma cell neoplasia and selected melanoma, prostate and mammary gland cancer cell lines [12,13].

In order to verify the role of DNA methylation within the CpG island of the *PHD3* gene, we treated HCT116 and DLD-1 cells with 5-dAzaC under normoxic and hypoxic conditions. 5-dAzaC was previously shown to induce the expression of many genes in different types of cancer and inhibit the growth of CRC cells [75,76]. We observed significant DNA demethylation in the chr14: 34 419 922–34 420 080, chr14: 34 419 795–34 419 935 and chr14: 34 419 400–34 419 538 regions of the CpG island of the *PHD3* gene in HCT116 cells incubated with 5-dAzaC, which was correlated with an increase in PHD3 transcript and protein levels. The same regions were unmethylated in DLD-1 cells at different experimental conditions and 5-dAzaC did not affect *PHD3* gene expression.

The presence of DNA hypermethylation of the *PHD3* promoter region in a broad range of human cancers suggests its role in tumour survival. In glioblastoma cell lines, accumulation of PHD3 protected tumour cells against hypoxia-induced cell death via control of HIF [77]. However, reduction of *PHD3* expression by DNA methylation may allow for stable HIF directed cellular response during hypoxia [12]. Moreover, in a subset of breast, prostate, melanoma and renal carcinoma cell lines, HIF-1α accumulation during hypoxia was independent of DNA hypermethylation of the *PHD3* promoter region, which suggests its role in other pathways and hydroxylase independent function [12].

## Conclusion

Our results showed increased DNA methylation levels in the CpG island of *PHD3* in CRC as compared to normal colonic epithelium from the same patients. These epigenetic alterations were associated with a significant decrease of PHD3 expression levels in patients with CRC. However, the reduction in *PHD1* and *PHD2* expression in cancerous tissue was not due to altered methylation within the CpG island in the promoter region of *PHD1* and *PHD2*, respectively. Therefore, other mechanisms might be responsible for the observed decreased expression levels of *PHD1* and *PHD2* in CRC patients. Furthermore, we observed an increased level of FIH protein in CRC, with no changes in the FIH transcript level between cancerous and histopathologically unchanged colonic mucosa. Additionally, we observed different statistical values of transcript and protein levels of the analyzed genes for subgroups of patients classified according to different features. However, a larger group of patient and deeper molecular investigation of these observations should be studied to determine whether the results within subgroups may be clinically important.

We also found that an inhibitor of DNMTs, 5-dAzaC, induced DNA demethylation of the *PHD3* promoter region, which was associated with increased transcript and protein levels in HCT116 cells under hypoxic and normoxic conditions.

Although we presented epigenetic transcriptional down-regulation of *PHD3* gene expression in CRC patients and HCT116 CRC cell line for the first time, further studies are required to verify and determine the role of CpG island methylation in *PHD3* expression in CRC to a greater extent. Moreover, DNA methylation is often associated with other alterations in chromatin structure, namely histone acetylation. High levels of DNA methylation accompanied with a low degree of histone acetylation may decrease the transcription of many genes [78]. In consideration of this, we also need to determine the possible role of histone modifications in *PHD3* gene expression.

## Competing interests

The authors declare that they have no competing interests.

## Authors’ contributions

AAR contributed to designing the study, acquisition of data, analysis and interpretation of data, and in writing the manuscript. KEB and KH participated in the acquisition and interpretation of the data. As Principal Investigators, PK and JPP were involved in the intellectual and experimental programming of the study, the interpretation of data, and writing the manuscript. All authors read and approved the final manuscript.

## Pre-publication history

The pre-publication history for this paper can be accessed here:

http://www.biomedcentral.com/1471-2407/13/526/prepub

## Supplementary Material

Additional file 1 Primer sequences. Click here for file

Additional file 2 **Schematic representation of the CpG distribution within the 5’ regulatory region of the *PHD1 *** **(A), *PHD2 *** **(B), *PHD3 *** **(C) and *FIH *** **(D) genes.** Click here for file

Additional file 3 Detection of heterogeneous methylation with HRM analysis. Click here for file

Additional file 4 PHD1 (A), PHD2 (B), PHD3 (C), FIH (D) transcript and protein levels in primary cancerous and histopathologically unchanged tissue samples from patients with CRC. Click here for file

Additional file 5 **Bisulfite sequencing of DNA CpG rich region of the *PHD1, PHD2 *** **and *FIH *** **genes in primary tissue samples from patients with CRC.** Click here for file

Additional file 6 **DNA methylation assessment of the *PHD1***, ***PHD2 *** **and *FIH *** **gene regulatory region by HRM analysis.** Click here for file

## References

[B1] Jemal A Siegel R Xu J Ward E Cancer statistics, 2010 CA Cancer J Clin 2010 60 5 277 300 10.3322/caac.20073 20610543

[B2] Webb JD Coleman ML Pugh CW Hypoxia, hypoxia-inducible factors (HIF), HIF hydroxylases and oxygen sensing Cell Mol Life Sci 2009 66 22 3539 3554 10.1007/s00018-009-0147-7 19756382 PMC11115642

[B3] Jokilehto T Jaakkola PM The role of HIF prolyl hydroxylases in tumour growth J Cell Mol Med 2010 14 4 758 770 10.1111/j.1582-4934.2010.01030.x 20178464 PMC3823110

[B4] Brahimi-Horn MC Bellot G Pouyssegur J Hypoxia and energetic tumour metabolism Curr Opin Genet Dev 2011 21 1 67 72 10.1016/j.gde.2010.10.006 21074987

[B5] Lando D Peet DJ Whelan DA Gorman JJ Whitelaw ML Asparagine hydroxylation of the HIF transactivation domain a hypoxic switch Science 2002 295 5556 858 861 10.1126/science.1068592 11823643

[B6] Lando D Peet DJ Gorman JJ Whelan DA Whitelaw ML Bruick RK FIH-1 is an asparaginyl hydroxylase enzyme that regulates the transcriptional activity of hypoxia-inducible factor Genes Dev 2002 16 12 1466 1471 10.1101/gad.991402 12080085 PMC186346

[B7] Soilleux EJ Turley H Tian YM Pugh CW Gatter KC Harris AL Use of novel monoclonal antibodies to determine the expression and distribution of the hypoxia regulatory factors PHD-1, PHD-2, PHD-3 and FIH in normal and neoplastic human tissues Histopathology 2005 47 6 602 610 10.1111/j.1365-2559.2005.02280.x 16324198

[B8] Xue J Li X Jiao S Wei Y Wu G Fang J Prolyl hydroxylase-3 is down-regulated in colorectal cancer cells and inhibits IKKbeta independent of hydroxylase activity Gastroenterology 2010 138 2 606 615 10.1053/j.gastro.2009.09.049 19786027

[B9] Xie G Zheng L Ou J Huang H He J Li J Pan F Liang H Low expression of prolyl hydroxylase 2 is associated with tumor grade and poor prognosis in patients with colorectal cancer Exp Biol Med (Maywood) 2012 237 7 860 866 10.1258/ebm.2012.011331 22802519

[B10] Jubb AM Turley H Moeller HC Steers G Han C Li JL Leek R Tan EY Singh B Mortensen NJ Expression of delta-like ligand 4 (Dll4) and markers of hypoxia in colon cancer Br J Cancer 2009 101 10 1749 1757 10.1038/sj.bjc.6605368 19844231 PMC2778546

[B11] Kondo Y Epigenetic cross-talk between DNA methylation and histone modifications in human cancers Yonsei Med J 2009 50 4 455 463 10.3349/ymj.2009.50.4.455 19718392 PMC2730606

[B12] Place TL Fitzgerald MP Venkataraman S Vorrink SU Case AJ Teoh ML Domann FE Aberrant promoter CpG methylation is a mechanism for impaired PHD3 expression in a diverse set of malignant cells PLoS One 2011 6 1 e14617 10.1371/journal.pone.0014617 21297970 PMC3030558

[B13] Hatzimichael E Dasoula A Shah R Syed N Papoudou-Bai A Coley HM Dranitsaris G Bourantas KL Stebbing J Crook T The prolyl-hydroxylase EGLN3 and not EGLN1 is inactivated by methylation in plasma cell neoplasia Eur J Haematol 2010 84 1 47 51 10.1111/j.1600-0609.2009.01344.x 19737309

[B14] Huang KT Mikeska T Dobrovic A Fox SB DNA methylation analysis of the HIF-1alpha prolyl hydroxylase domain genes PHD1, PHD2, PHD3 and the factor inhibiting HIF gene FIH in invasive breast carcinomas Histopathology 2010 57 3 451 460 10.1111/j.1365-2559.2010.03633.x 20727020

[B15] Durczak M Jagodzinski PP Apicidin upregulates PHD2 prolyl hydroxylase gene expression in cervical cancer cells Anticancer Drugs 2010 21 6 619 624 10.1097/CAD.0b013e328339848b 20527723

[B16] Espina V Mueller C Edmiston K Sciro M Petricoin EF Liotta LA Tissue is alive: new technologies are needed to address the problems of protein biomarker pre-analytical variability Proteomics 2009 3 8 874 882 20871745 10.1002/prca.200800001 PMC2944287

[B17] Chomczynski P Sacchi N Single-step method of RNA isolation by acid guanidinium thiocyanate-phenol-chloroform extraction Anal Biochem 1987 162 1 156 159 2440339 10.1006/abio.1987.9999

[B18] Vandesompele J De Preter K Pattyn F Poppe B Van Roy N De Paepe A Speleman F Accurate normalization of real-time quantitative RT-PCR data by geometric averaging of multiple internal control genes Genome Biol 2002 3 7 RESEARCH0034 12184808 10.1186/gb-2002-3-7-research0034 PMC126239

[B19] Sorby LA Andersen SN Bukholm IR Jacobsen MB Evaluation of suitable reference genes for normalization of real-time reverse transcription PCR analysis in colon cancer J Exp Clin Cancer Res 2010 29 144 10.1186/1756-9966-29-144 21059236 PMC2988724

[B20] EMBOSS CpGPlot/CpGReport/Isochore http://www.ebi.ac.uk/emboss/cpgplot/

[B21] CpG Island Searcher http://cpgislands.usc.edu/

[B22] UCSC Genome Bioinformatics Site http://genome.ucsc.edu/

[B23] Bock C Reither S Mikeska T Paulsen M Walter J Lengauer T BiQ Analyzer: visualization and quality control for DNA methylation data from bisulfite sequencing Bioinformatics 2005 21 21 4067 4068 10.1093/bioinformatics/bti652 16141249

[B24] Rohde C Zhang Y Jurkowski TP Stamerjohanns H Reinhardt R Jeltsch A Bisulfite sequencing Data Presentation and Compilation (BDPC) web server–a useful tool for DNA methylation analysis Nucleic Acids Res 2008 36 5 e34 10.1093/nar/gkn083 18296484 PMC2275153

[B25] Wojdacz TK Dobrovic A Methylation-sensitive high resolution melting (MS-HRM): a new approach for sensitive and high-throughput assessment of methylation Nucleic Acids Res 2007 35 6 e41 10.1093/nar/gkm013 17289753 PMC1874596

[B26] Rawluszko AA Horbacka K Krokowicz P Jagodzinski PP Decreased expression of 17-beta-hydroxysteroid dehydrogenase type 1 is associated with DNA hypermethylation in colorectal cancer located in the proximal colon BMC Cancer 2011 11 1 522 10.1186/1471-2407-11-522 22176788 PMC3280200

[B27] Hsieh CL Dependence of transcriptional repression on CpG methylation density Mol Cell Biol 1994 14 8 5487 5494 7518564 10.1128/mcb.14.8.5487-5494.1994 PMC359068

[B28] Shao Y Zhang W Zhang C Wu Q Yang H Zhang J Guan M Wan J Yu B High-resolution melting analysis of BLU methylation levels in gastric, colorectal, and pancreatic cancers Cancer Invest 2010 28 6 642 648 10.3109/07357901003631023 20394502

[B29] Dimitrakopoulos L Vorkas PA Georgoulias V Lianidou ES A closed-tube methylation-sensitive high resolution melting assay (MS-HRMA) for the semi-quantitative determination of CST6 promoter methylation in clinical samples BMC Cancer 2012 12 486 10.1186/1471-2407-12-486 23088560 PMC3495665

[B30] Oster B Thorsen K Lamy P Wojdacz TK Hansen LL Birkenkamp-Demtroder K Sorensen KD Laurberg S Orntoft TF Andersen CL Identification and validation of highly frequent CpG island hypermethylation in colorectal adenomas and carcinomas Int J Cancer 2011 129 12 2855 2866 10.1002/ijc.25951 21400501

[B31] Dukes CE Bussey HJ The spread of rectal cancer and its effect on prognosis Br J Cancer 1958 12 3 309 320 10.1038/bjc.1958.37 13596482 PMC2073915

[B32] Brahimi-Horn MC Chiche J Pouyssegur J Hypoxia and cancer J Mol Med 2007 85 12 1301 1307 10.1007/s00109-007-0281-3 18026916

[B33] Adams GE Dische S Fowler JF Thomlinson RH Hypoxic cell sensitisers in radiotherapy Lancet 1976 1 7952 186 188 54693 10.1016/s0140-6736(76)91285-x

[B34] Comerford KM Wallace TJ Karhausen J Louis NA Montalto MC Colgan SP Hypoxia-inducible factor-1-dependent regulation of the multidrug resistance (MDR1) gene Cancer Res 2002 62 12 3387 3394 12067980

[B35] Appelhoff RJ Tian YM Raval RR Turley H Harris AL Pugh CW Ratcliffe PJ Gleadle JM Differential function of the prolyl hydroxylases PHD1, PHD2, and PHD3 in the regulation of hypoxia-inducible factor J Biol Chem 2004 279 37 38458 38465 10.1074/jbc.M406026200 15247232

[B36] Couvelard A Deschamps L Rebours V Sauvanet A Gatter K Pezzella F Ruszniewski P Bedossa P Overexpression of the oxygen sensors PHD-1, PHD-2, PHD-3, and FIH Is associated with tumor aggressiveness in pancreatic endocrine tumors Clin Cancer Res 2008 14 20 6634 6639 10.1158/1078-0432.CCR-07-5258 18927305

[B37] Boddy JL Fox SB Han C Campo L Turley H Kanga S Malone PR Harris AL The androgen receptor is significantly associated with vascular endothelial growth factor and hypoxia sensing via hypoxia-inducible factors HIF-1a, HIF-2a, and the prolyl hydroxylases in human prostate cancer Clin Cancer Res 2005 11 21 7658 7663 10.1158/1078-0432.CCR-05-0460 16278385

[B38] Andersen S Donnem T Stenvold H Al-Saad S Al-Shibli K Busund LT Bremnes RM Overexpression of the HIF hydroxylases PHD1, PHD2, PHD3 and FIH are individually and collectively unfavorable prognosticators for NSCLC survival PLoS One 2011 6 8 e23847 10.1371/journal.pone.0023847 21887331 PMC3161788

[B39] Zhang Q Gu J Li L Liu J Luo B Cheung HW Boehm JS Ni M Geisen C Root DE Control of cyclin D1 and breast tumorigenesis by the EglN2 prolyl hydroxylase Cancer Cell 2009 16 5 413 424 10.1016/j.ccr.2009.09.029 19878873 PMC2788761

[B40] Erez N Milyavsky M Eilam R Shats I Goldfinger N Rotter V Expression of prolyl-hydroxylase-1 (PHD1/EGLN2) suppresses hypoxia inducible factor-1alpha activation and inhibits tumor growth Cancer Res 2003 63 24 8777 8783 14695194

[B41] Peurala E Koivunen P Bloigu R Haapasaari KM Jukkola-Vuorinen A Expressions of individual PHDs associate with good prognostic factors and increased proliferation in breast cancer patients Breast Cancer Res Treat 2012 133 1 179 188 10.1007/s10549-011-1750-5 21877141

[B42] Jokilehto T Rantanen K Luukkaa M Heikkinen P Grenman R Minn H Kronqvist P Jaakkola PM Overexpression and nuclear translocation of hypoxia-inducible factor prolyl hydroxylase PHD2 in head and neck squamous cell carcinoma is associated with tumor aggressiveness Clin Cancer Res 2006 12 4 1080 1087 10.1158/1078-0432.CCR-05-2022 16489060

[B43] Jokilehto T Hogel H Heikkinen P Rantanen K Elenius K Sundstrom J Jaakkola PM Retention of prolyl hydroxylase PHD2 in the cytoplasm prevents PHD2-induced anchorage-independent carcinoma cell growth Exp Cell Res 2010 316 7 1169 1178 10.1016/j.yexcr.2010.02.012 20156434

[B44] Gossage L Zaitoun A Fareed KR Turley H Aloysius M Lobo DN Harris AL Madhusudan S Expression of key hypoxia sensing prolyl-hydroxylases PHD1, -2 and -3 in pancreaticobiliary cancer Histopathology 2010 56 7 908 920 10.1111/j.1365-2559.2010.03566.x 20497244

[B45] Hogel H Rantanen K Jokilehto T Grenman R Jaakkola PM Prolyl hydroxylase PHD3 enhances the hypoxic survival and G1 to S transition of carcinoma cells PLoS One 2011 6 11 e27112 10.1371/journal.pone.0027112 22087251 PMC3210766

[B46] Su C Huang K Sun L Yang D Zheng H Gao C Tong J Zhang Q Overexpression of the HIF hydroxylase PHD3 is a favorable prognosticator for gastric cancer Med Oncol 2012 10.1007/s12032-012-0171-6 22290580

[B47] Lee S Nakamura E Yang H Wei W Linggi MS Sajan MP Farese RV Freeman RS Carter BD Kaelin WG Jr Neuronal apoptosis linked to EglN3 prolyl hydroxylase and familial pheochromocytoma genes: developmental culling and cancer Cancer Cell 2005 8 2 155 167 10.1016/j.ccr.2005.06.015 16098468

[B48] Schlisio S Kenchappa RS Vredeveld LC George RE Stewart R Greulich H Shahriari K Nguyen NV Pigny P Dahia PL The kinesin KIF1Bbeta acts downstream from EglN3 to induce apoptosis and is a potential 1p36 tumor suppressor Genes Dev 2008 22 7 884 893 10.1101/gad.1648608 18334619 PMC2279200

[B49] Rantanen K Pursiheimo J Hogel H Himanen V Metzen E Jaakkola PM Prolyl hydroxylase PHD3 activates oxygen-dependent protein aggregation Mol Biol Cell 2008 19 5 2231 2240 10.1091/mbc.E07-11-1124 18337469 PMC2366864

[B50] D'Angelo G Duplan E Boyer N Vigne P Frelin C Hypoxia up-regulates prolyl hydroxylase activity: a feedback mechanism that limits HIF-1 responses during reoxygenation J Biol Chem 2003 278 40 38183 38187 10.1074/jbc.M302244200 12876291

[B51] Takeda K Ho VC Takeda H Duan LJ Nagy A Fong GH Placental but not heart defects are associated with elevated hypoxia-inducible factor alpha levels in mice lacking prolyl hydroxylase domain protein 2 Mol Cell Biol 2006 26 22 8336 8346 10.1128/MCB.00425-06 16966370 PMC1636770

[B52] Fu J Menzies K Freeman RS Taubman MB EGLN3 prolyl hydroxylase regulates skeletal muscle differentiation and myogenin protein stability J Biol Chem 2007 282 17 12410 12418 10.1074/jbc.M608748200 17344222

[B53] Dayan F Roux D Brahimi-Horn MC Pouyssegur J Mazure NM The oxygen sensor factor-inhibiting hypoxia-inducible factor-1 controls expression of distinct genes through the bifunctional transcriptional character of hypoxia-inducible factor-1alpha Cancer Res 2006 66 7 3688 3698 10.1158/0008-5472.CAN-05-4564 16585195

[B54] Pelletier J Dayan F Durivault J Ilc K Pecou E Pouyssegur J Mazure NM The asparaginyl hydroxylase factor-inhibiting HIF is essential for tumor growth through suppression of the p53-p21 axis Oncogene 2012 31 24 2989 3001 10.1038/onc.2011.471 22002313

[B55] Mahon PC Hirota K Semenza GL FIH-1: a novel protein that interacts with HIF-1alpha and VHL to mediate repression of HIF-1 transcriptional activity Genes Dev 2001 15 20 2675 2686 10.1101/gad.924501 11641274 PMC312814

[B56] Cockman ME Lancaster DE Stolze IP Hewitson KS McDonough MA Coleman ML Coles CH Yu X Hay RT Ley SC Posttranslational hydroxylation of ankyrin repeats in IkappaB proteins by the hypoxia-inducible factor (HIF) asparaginyl hydroxylase, factor inhibiting HIF (FIH) Proc Natl Acad Sci U S A 2006 103 40 14767 14772 10.1073/pnas.0606877103 17003112 PMC1578504

[B57] Coleman ML McDonough MA Hewitson KS Coles C Mecinovic J Edelmann M Cook KM Cockman ME Lancaster DE Kessler BM Asparaginyl hydroxylation of the Notch ankyrin repeat domain by factor inhibiting hypoxia-inducible factor J Biol Chem 2007 282 33 24027 24038 10.1074/jbc.M704102200 17573339

[B58] Ferguson JE 3rd Wu Y Smith K Charles P Powers K Wang H Patterson C ASB4 is a hydroxylation substrate of FIH and promotes vascular differentiation via an oxygen-dependent mechanism Mol Cell Biol 2007 27 18 6407 6419 10.1128/MCB.00511-07 17636018 PMC2099627

[B59] Fukuba H Takahashi T Jin HG Kohriyama T Matsumoto M Abundance of aspargynyl-hydroxylase FIH is regulated by Siah-1 under normoxic conditions Neurosci Lett 2008 433 3 209 214 10.1016/j.neulet.2007.12.069 18280659

[B60] Hu G Fearon ER Siah-1 N-terminal RING domain is required for proteolysis function, and C-terminal sequences regulate oligomerization and binding to target proteins Mol Cell Biol 1999 19 1 724 732 9858595 10.1128/mcb.19.1.724 PMC83929

[B61] Bruzzoni-Giovanelli H Fernandez P Veiga L Podgorniak M-P Powell D Candeias M Mourah S Calvo F Marin M Distinct expression patterns of the E3 ligase SIAH-1 and its partner Kid/KIF22 in normal tissues and in the breast tumoral processes J Exp Clin Cancer Res 2010 29 1 10 10.1186/1756-9966-29-10 20144232 PMC2831832

[B62] Matsuo K Satoh S Okabe H Nomura A Maeda T Yamaoka Y Ikai I SIAH1 inactivation correlates with tumor progression in hepatocellular carcinomas Genes Chromosomes Cancer 2003 36 3 283 291 10.1002/gcc.10170 12557228

[B63] Wen YY Yang ZQ Song M Li BL Yao XH Chen XL Zhao J Lu YY Zhu JJ Wang EH The expression of SIAH1 is downregulated and associated with Bim and apoptosis in human breast cancer tissues and cells Mol Carcinog 2010 49 5 440 449 20082325 10.1002/mc.20615

[B64] Kim YI Nutritional epigenetics: impact of folate deficiency on DNA methylation and colon cancer susceptibility J Nutr 2005 135 11 2703 2709 16251634 10.1093/jn/135.11.2703

[B65] Hermann S Rohrmann S Linseisen J Lifestyle factors, obesity and the risk of colorectal adenomas in EPIC-Heidelberg Cancer Causes Control 2009 20 8 1397 1408 10.1007/s10552-009-9366-3 19466571

[B66] Fearon ER Vogelstein B A genetic model for colorectal tumorigenesis Cell 1990 61 5 759 767 10.1016/0092-8674(90)90186-I 2188735

[B67] Kim MS Lee J Sidransky D DNA methylation markers in colorectal cancer Cancer Metastasis Rev 2010 29 1 181 206 10.1007/s10555-010-9207-6 20135198

[B68] Chan AO Broaddus RR Houlihan PS Issa JP Hamilton SR Rashid A CpG island methylation in aberrant crypt foci of the colorectum Am J Pathol 2002 160 5 1823 1830 10.1016/S0002-9440(10)61128-5 12000733 PMC1850869

[B69] Suehiro Y Hinoda Y Genetic and epigenetic changes in aberrant crypt foci and serrated polyps Cancer Sci 2008 99 6 1071 1076 10.1111/j.1349-7006.2008.00784.x 18384435 PMC11159269

[B70] Kane MF Loda M Gaida GM Lipman J Mishra R Goldman H Jessup JM Kolodner R Methylation of the hMLH1 promoter correlates with lack of expression of hMLH1 in sporadic colon tumors and mismatch repair-defective human tumor cell lines Cancer Res 1997 57 5 808 811 9041175

[B71] Veigl ML Kasturi L Olechnowicz J Ma AH Lutterbaugh JD Periyasamy S Li GM Drummond J Modrich PL Sedwick WD Biallelic inactivation of hMLH1 by epigenetic gene silencing, a novel mechanism causing human MSI cancers Proc Natl Acad Sci U S A 1998 95 15 8698 8702 10.1073/pnas.95.15.8698 9671741 PMC21139

[B72] Herman JG Merlo A Mao L Lapidus RG Issa JP Davidson NE Sidransky D Baylin SB Inactivation of the CDKN2/p16/MTS1 gene is frequently associated with aberrant DNA methylation in all common human cancers Cancer Res 1995 55 20 4525 4530 7553621

[B73] Pescador N Cuevas Y Naranjo S Alcaide M Villar D Landazuri MO Del Peso L Identification of a functional hypoxia-responsive element that regulates the expression of the egl nine homologue 3 (egln3/phd3) gene Biochem J 2005 390 Pt 1 189 197 15823097 10.1042/BJ20042121 PMC1184574

[B74] Shahrzad S Bertrand K Minhas K Coomber BL Induction of DNA hypomethylation by tumor hypoxia Epigenetics 2007 2 2 119 125 10.4161/epi.2.2.4613 17965619

[B75] Momparler RL Pharmacology of 5-Aza-2'-deoxycytidine (decitabine) Semin Hematol 2005 42 3 Suppl 2 S9 S16 16015507 10.1053/j.seminhematol.2005.05.002

[B76] Ghoshal K Motiwala T Claus R Yan P Kutay H Datta J Majumder S Bai S Majumder A Huang T HOXB13, a target of DNMT3B, is methylated at an upstream CpG island, and functions as a tumor suppressor in primary colorectal tumors PLoS One 2010 5 4 e10338 10.1371/journal.pone.0010338 20454457 PMC2861599

[B77] Henze AT Riedel J Diem T Wenner J Flamme I Pouyseggur J Plate KH Acker T Prolyl hydroxylases 2 and 3 act in gliomas as protective negative feedback regulators of hypoxia-inducible factors Cancer Res 2010 70 1 357 366 10.1158/0008-5472.CAN-09-1876 20028863

[B78] Luczak MW Jagodzinski PP The role of DNA methylation in cancer development Folia Histochem Cytobiol 2006 44 3 143 154 16977793

