# Supplementary material for: Expression and DNA methylation levels of prolyl hydroxylases PHD1, PHD2, PHD3 and asparaginyl hydroxylase FIH in colorectal cancer
Source: BMC Cancer. 2013 Nov 6;13:526. doi: 10.1186/1471-2407-13-526 (PMC3828400; doi:10.1186/1471-2407-13-526)
Supplement: Additional file 1 — Primer sequences. [file 1471-2407-13-526-S1.pdf]

Gene	Sequence (5’-3’)	UCSC position (GRCh37/hg19)	Product size (bp)	Method
PHD1	F: TATGCCACCAGGTACGCCATC<br>R: AGGAGCAGCCAGCACAGG	chr19: 41 313 169-41 313 844	210	RT-PCR
PHD2	F: GGACGACCTGATACG<br>R: ACTTACCTTGGCATCC	chr1: 231 506424-231 556 792	190	RT-PCR
PHD3	F: CCTCTTACGCAACCAGATATG<br>R: AGCACGGTCAGTCTTCAG	chr14: 34 395 090-34 398 297	129	RT-PCR
FIH	F: ACAGTGCCAGCACCCACAA<br>R: GCCCACAGTGTCATTGAGCG	chr10:102 296 208-102 300 427	188	RT-PCR
PBDG	F: GCCAAGGACCAGGACATC<br>R: TCAGGTACAGTTGCCCATC	chr11:118 468 348-118 468 864	160	RT-PCR
hMRPL19	F: ACTTTATAATCCTCGGGTC<br>R: ACTTTCAGCTCATTAACAG	chr2:75 735 389- 75 735 705	171	RT-PCR
PHD1S	F: TATGAATTTTGTGTGTGTTTTGA<br>R: TATGAATTTTGTGTGTGTTTTGA	chr19: 41 304 043-41 304 698	656	BS
PHD2S1	F: TGGAGGAAGAGTAGTTATG<br>R: ATAACAACCTAAACTCAAAC	chr1: 231 557 896-231 558 421	526	BS
PHD2S2	F: GTTTGAGTTTAGGTTGTTATT<br>R: AATACTAATATAAACCCACTC	chr1:231 557 412-231 557 915	504	BS
PHD3S1	F: AGTGTGGGATTTAGGTTTTTAAGT	chr14:34 419 929-34 420 563	635	BS

	R: ATCCAACCTCATAATATATCCCA			
PHD3S2	F: ATTATGAGGTTGGATTTGGAGA<br>R: ATCACCCACAAAAACCAATATT	ch14: 34 419 346-34 419 943	598	BS
FIHS1	F: TGAATTTGTTTTTTTTTTTGA<br>R: ACCAAAAACCACAACCTC	chr10: 102 295 385–102 299 751	367	BS
PHD1.1	F: GTGTGGTTATGAATTTTGTGTG<br>R: CTACACCAATCCCCTACAAAC	chr19: 41 304 036-41 304 177	142	HRMA
PHD1.2	F: CGGGTAGGTTTGTTTATGTAAA<br>R: ACACGAAATTCATTTACATCCT	chr19: 41 304 758-41 304 922	165	HRMA
PHD2.1	F: GGGAGTTTGATTTTTGGATTTAGT<br>R: AATCATAACTACTCTTCCTCCAAACC	chr1: 231 558 400 – 231 558 576	177	HRMA
PHD2.2	F: TTAGCGTTAGGATTGGAAGAAG<br>R: ACCTTCCTAAACTCCCGAAC	chr1: 231 557 348- 231 557 497	150	HRMA
PHD2.3	F:GGGGTTCGGGAGTTTAGGAAGGTAG<br>R: CCGAAAACTTAACCTTTACTTTTCCCTTAAC	chr1:231 557 283-231 557 370	88	HRMA
PHD3.1	F: CGTGGTAGTCGTAGGTTTTTG<br>R: TCTCCAAATCCAACCTCATAAT	ch14: 34 419 922-34 420 080	159	HRMA
PHD3.2	F: GTTGGATTTGGAGAAAATTG<br>R: ACCCCGATACAATACAACTAC	ch14: 34 419 795-34 419 935	141	HRMA
PHD3.3	F: TTGTATGTCGATTTTTTAGGGT<br>R: ACCTAAACCCCTTAACGTTAAC	chr14: 34 419 400 -34 419 538	139	HRMA

FIH.1	F: TTGGGTGTGTGGTTTTTTTG<br>R: CGAAACTCTCCAAAACCAAAAACC	chr10: 102 295 626- 102 295 765	140	HRMA

Additional file 1. Primer sequences

RT-PCR- real-time quantitative polymerase chain reaction

BS- bisulfite sequencing

HRMA- high resolution melting analysis

Primer localization for BS and HRMA was presented in Additional file 2.
