# Supplementary material for: Expression and DNA methylation levels of prolyl hydroxylases PHD1, PHD2, PHD3 and asparaginyl hydroxylase FIH in colorectal cancer
Source: BMC Cancer. 2013 Nov 6;13:526. doi: 10.1186/1471-2407-13-526 (PMC3828400; doi:10.1186/1471-2407-13-526)
Supplement: Additional file 2 — Schematic representation of the CpG distribution within the 5’ regulatory region of the PHD1 (A), PHD2 (B), PHD3 (C) and FIH (D) genes. [file 1471-2407-13-526-S2.pdf]

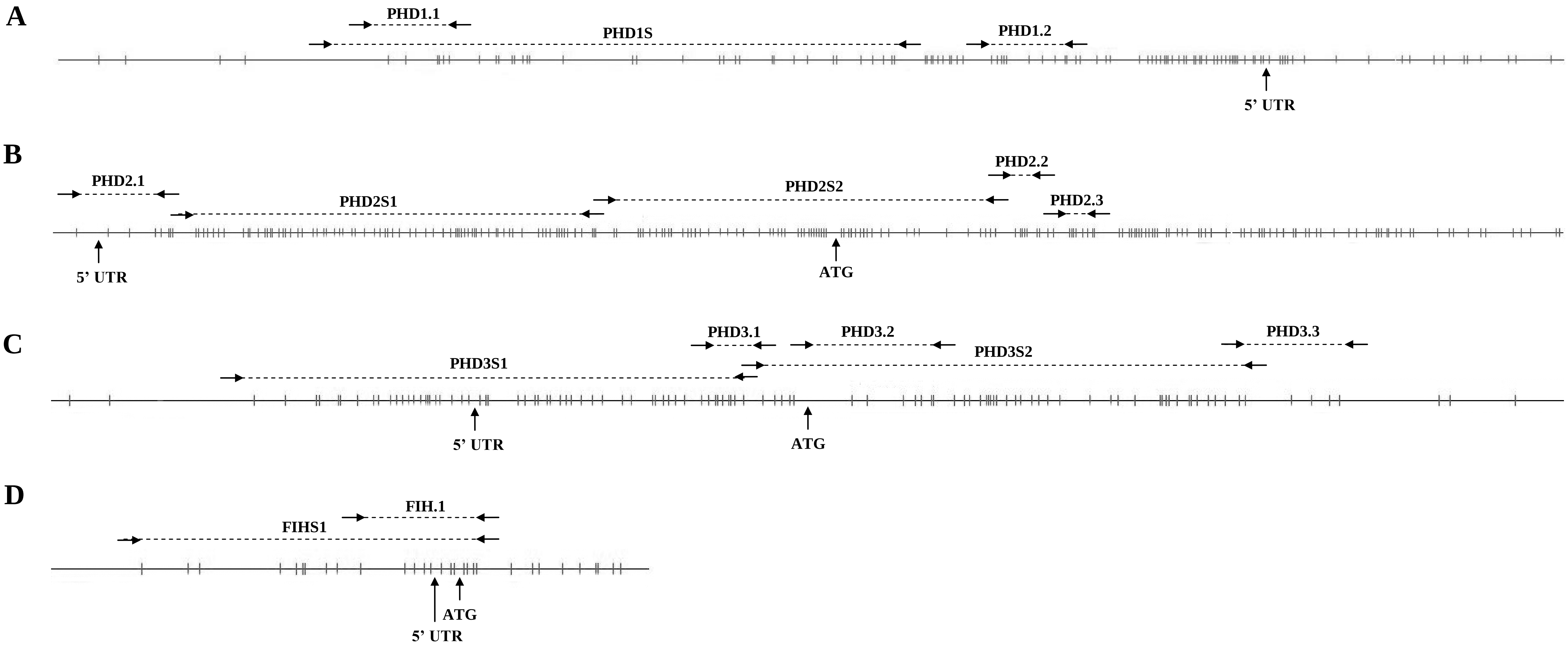

Additional file 2. Schematic representation of the CpG distribution within 5' regulatory region of the *PHD1* (A), *PHD2* (B), *PHD3* (C) and *FIH* (D) gene. The CpG dinucleotides are represented by vertical tick marks and the position of 5'UTR and ATG sites are depicted with arrow. Horizontal arrows with dotted lane correspond to the position of primers and PCR product, respectively used for the bisulfite sequencing and HRM analysis.
