# Supplementary material for: Expression and DNA methylation levels of prolyl hydroxylases PHD1, PHD2, PHD3 and asparaginyl hydroxylase FIH in colorectal cancer
Source: BMC Cancer. 2013 Nov 6;13:526. doi: 10.1186/1471-2407-13-526 (PMC3828400; doi:10.1186/1471-2407-13-526)
Supplement: Additional file 3 — Detection of heterogeneous methylation with HRM analysis. [file 1471-2407-13-526-S3.pdf]

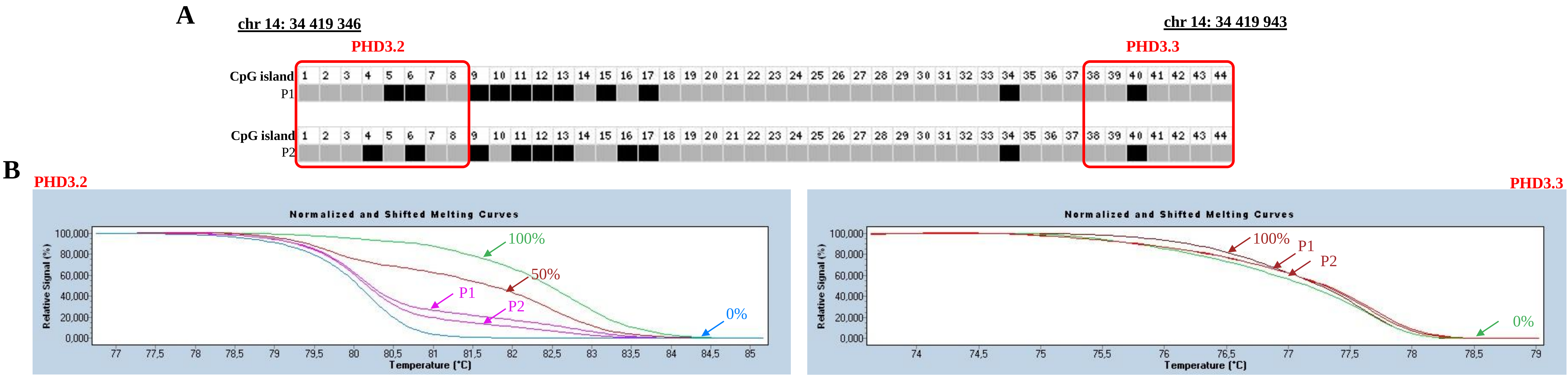


**Additional file 6. Detection of heterogeneous methylation with HRM analysis. A.** Primary cancerous tissues (P1, P2) were used for genomic DNA isolation followed by bisulfite conversion of cytosine to uracil. The *PHD3* regions containing 44 CpG dinucleotides (ch14: 34 419 346-34 419 943) was then amplified by a pair of primers complementary to the bisulfite-DNA modified sequence (Additional file 1, Additional file 2). The PCR product were purified with subsequent cloning into a plasmid vector. Plasmid DNA isolated from positive bacterial clones was used for commercial sequencing. The results of bisulfite sequencing were assessed and presented using BiQ analyzer software and BDPC web server [23, 24]. Black and grey boxes represent methylated and unmethylated CpG dinucleotide, respectively. Red rectangles correspond to regions amplified in HRM analysis by specific primers PHD3.2 and PHD3.3 (Additional file 1, Additional file 2). **B.** represents HRM profiles of standard and plasmid DNA used in bisulfite sequencing (P1, P2). DNA standards were prepared by mixing different ratios of methylated and non-methylated bisulfite treated DNA. HRM methylation analysis was performed using Light Cycler®480 Gene Scanning software, Roche Diagnostics GmbH (Mannheim, Germany). Each PCR amplification and HRM profile analysis was performed in triplicate.
