# Supplementary material for: Expression and DNA methylation levels of prolyl hydroxylases PHD1, PHD2, PHD3 and asparaginyl hydroxylase FIH in colorectal cancer
Source: BMC Cancer. 2013 Nov 6;13:526. doi: 10.1186/1471-2407-13-526 (PMC3828400; doi:10.1186/1471-2407-13-526)
Supplement: Additional file 4 — PHD1 (A), PHD2 (B), PHD3 (C), FIH (D) transcript and protein levels in primary cancerous and histopathologically unchanged tissue samples from patients with CRC. [file 1471-2407-13-526-S4.pdf]

Additional file 3. PHD1 (A), PHD2 (B), PHD3 (C), FIH (D) transcript and protein levels in primary cancerous and histopathologically unchanged tissue samples from patient with CRC.

A.

PHD1		primary cancerous tissues median (range)	histopathologically unchanged tissues median (range)	p value
	transcript	3.278 (1.240-.738)	3.515 (1.459-5.204)	0.000296
	protein	2.612 (1.132-3.560)	2.968 (2.132-5.256)	0.00416
**Age (Years)**				
<60	transcript	3.264 (2.854-4.738)	3.394 (2.784-5.204)	0.0387
	protein	2.549 (1.805-3.414)	3.000 (2.579-3.512)	0.0576
>60	transcript	3.303 (1.240-3.904)	3.758 (1.459-4.896)	0.00428
	protein	2.612 (1.132-3.560)	2.962 (2.132-5.256)	0.0279
**Gender**				
Female	transcript	3.303 (1.241-3.904)	3.758 (1.459-4.896)	0.00189
	protein	2.707 (1.779-3.414)	3.000 (2.132-5.256)	0.0227
Male	transcript	3.264 (2.854-4.738)	3.394 (2.784-5.204)	0.0387
	protein	2.503 (1.132-3.560)	2.919 (2.152-4.011)	0.063
**CRC localization**				
Proximal colon (cecum to transverse)	transcript	3.246 (2.790-3.899)	3.351 (2.905-4.318)	0.176
	protein	2.838 (1.780-3.560)	3.139 (2.132-5.256)	0.0137
Distal colon (splenic flexture to sigmoid)	transcript	3.151 (2.854-3.669)	3.506 (2.784-4.328)	0.00548
	protein	2.480 (2.167-2.549)	2.896 (2.470-3.001)	0.0374
Rectum	transcript	3.344 (1.240-4.388)	3.549 (1.459-5.204)	0.0641
	protein	2.563 (1.132-3.414)	2.846 (2.152-3.704)	0.0991
**Histologic grade**				
G1	transcript	3.176 (3.157-3.266)	3.533 (3.122-4.318)	0.513
	protein	2.207 (2.065-2.350)	2.462 (2.152-2.772)	0.438
G2	transcript	3.278 (1.240-4.388)	3.462 (1.459-5.204)	0.0141
	protein	2.549 (1.132-3.413)	2.999 (2.316-4.848)	0.0105
G3	transcript	3.307 (2.854-3.754)	3.568 (3.029-4.896)	0.0136
	protein	2.612 (2.623-3.414)	2.885 (2.132-5.256)	0.521
**Dukes Scale**				
A	transcript	3.276 (3.157-3.669)	3.872 (3.218-4.318)	0.0476
	protein	2.543 (2.480-2.817)	2.470 (2.316-4.848)	0.513
B	transcript	3.305 (2.790-4.388)	3.487 (2.905-4.318)	0.213
	protein	2.549 (1.132-3.216)	3.000 (2.442-3.358)	0.0649
C	transcript	3.344 (1.240-3.899)	3.691 (1.459-5.204)	0.0115
	protein	2.744 (2.112-3.414)	2.954 (2.132-5.256)	0.0932
**Tumour stage**				
T1	transcript	3.279 (3.157-3.462)	3.872 (3.364-4.318)	0.127
	protein	2.412 (1.921-2.498)	2.951 (0.939-3.020)	0.517
T2	transcript	3.508 (3.231-3.697)	3.567 (3.216-4.238)	0.655
	protein	2.836 (2.480-2.982)	2.986 (2.470-4.848)	0.1745
T3	transcript	3.303 (1.240-4.388)	3.564 (1.459-5.204)	0.00747
	protein	2.600 (1.132-3.414)	2.968 (2.132-5.256)	0.0588
T4	transcript	3.326 (3.124-3.767)	3.644 (3.176-4.218)	0.337
	protein	2.437 (2.280-2.986)	2.951 (2.582-3.064)	0.275

B.

PHD2		primary cancerous tissues median (range)	histopathologically unchanged tissues median (range)	p value
	transcript	3.069 (1.842-.4.147)	3.355 (2.561-4.552)	p<0.00001
	protein	2.661 (0.374-4.132)	2.876 (1.749-5.545)	0.00710
Age (Years)				
<60	transcript	3.070 (2.727-3.460)	3.361 (3.012-3.784)	0.0243
	protein	2.605 (0.374-3.188)	3.032 (2.337-4.867)	0.000573
>60	transcript	3.058 (1.842-4.174)	3.354 (2.561-4.552)	0.000005
	protein	2.660 (0.374-3.189)	2.813 (1.749-4.867)	0.0230
Gender				
Female	transcript	3.042 (2.591-3.460)	3.438 (2.561-4.552)	0.000028
	protein	2.660 (1.616-3.077)	3.079 (2.157-5.545)	0.050
Male	transcript	3.088 (1.842-4.174)	3.264 (2.595-4.505)	0.00254
	protein	2.606 (0.374-3.188)	3.022 (2.337-4.867)	0.000573
CRC localization				
Proximal colon (cecum to transverse)	transcript	3.035 (2.058-3.747)	3.360 (2.724-4.552)	0.00611
	protein	2.534 (0.374-2.730)	2.721(2.157-3.439)	0.0364
Distal colon (splenic flexture to sigmoid)	transcript	2.974 (2.727-3.515)	3.413 (2.561-4.129)	0.00345
	protein	2.846 (2.469-3.188)	3.227 (3.044-3.783)	0.0281
Rectum	transcript	3.108 (1.842-4.147)	3.237 (2.596-4.506)	0.002167
	protein	2.678 (1.109-4.132)	3.051 (1.749-5.545)	0.0188
Histologic grade				
G1	transcript	2.862 (2.770-3.198)	3.447 (2.973-4.129)	0.0833
	protein	2.000 (0.374-2.900)	2.700 (2.400-2.800)	0.0615
G2	transcript	3.054 (1.842-4.147)	3.230 (2.561-4.552)	0.001285
	protein	2.669 (1.108-4.132)	2.876 (2.116-5.545)	0.0415
G3	transcript	3.105 (2.591-3.515)	3.414 (3.049-4.113)	0.000389
	protein	2.574 (1.251-3.188)	3.104 (1.749-4.867)	0.0576
Dukes Scale				
A	transcript	3.172 (2.905-3.515)	3.794 (2.982-4.552)	0.0587
	protein	2.100 (0.374- 2.300)	2.701 (2.402- 2.803)	0.169
B	transcript	3.105 (2.727-4.147)	3.319 (2.561-3.741)	0.0707
	protein	2.673 (2.209-3.188)	2.831 (2.157-3.915)	0.7728
C	transcript	3.069 (1.842-3.439)	3.385 (2.796-4.506)	0.000327
	protein	2.661 (1.108-4.132)	3.094 (1.749-5.545)	0.00634
Tumour stage				
T1	transcript	3.172 (2.905-3.404)	3.492 (2.982-4.129)	0.248
	protein	2.229 (0.374-2.568)	2.458 (1.749-2.786)	0.462
T2	transcript	3.181 (3.009-3.515)	3.414 (3.122-4.552)	0.0639
	protein	2.568 (2.229-4.132)	2.749 (2.458-2.786)	0.628
T3	transcript	3.036 (2.271-4.147)	3.348 (2.561-4.505)	0.000147
	protein	2.667 (1.107-3.414)	3.065 (2.157-5.545)	0.0294
T4	transcript	3.273 (1.842-3.435)	3.411 (2.880-3.764)	0.0782
	protein	2.751 (1.616-3.011)	3.099 (2.685-3.120)	0.275

C.

PHD3		primary cancerous tissues median (range)	histopathologically unchanged tissues median (range)	p value
	transcript	2.562 (1.941-4.245)	2.910 (2.005-5.062)	p<0.00001
	protein	2.481(0.677-3.590)	3.175 (0.939-5.518)	p<0.00001
**Age (Years)**				
<60	transcript	2.634 (2.018-3.085)	3.378 (2.561-4.734)	0.00791
	protein	2.505 (1.229-3.069)	3.227 (2.885-3.564)	0.00601
>60	transcript	2.534 (1.941-4.245)	2.901 (2.005-5.062)	0.000016
	protein	2.464 (0.677-3.590)	3.121 (0.939-5.518)	0.00001
**Gender**				
Female	transcript	2.617 (2.008-3.085)	2.931 (2.005-5.062)	0.000189
	protein	2.481 (1.229-3.590)	3.210 (0.939-4.638)	0.000665
Male	transcript	2.531 (1.941-4.245)	2.909 (2.233-4.734)	0.000832
	protein	2.507 (0.677-3.533)	2.986 (2.484-5.518)	0.00027
**CRC localization**				
Proximal colon (cecum to transverse)	transcript	2.556 (2.267-3.301)	2.794 (2.005-5.062)	0.00536
	protein	2.756 (1.395-3.590)	3.358 (1.978-4.638)	0.001725
Distal colon (splenic flexture to sigmoid)	transcript	2.699 (2.023-3.156)	2.960 (2.329-3.665)	0.0378
	protein	2.360 (1.538-2.604)	2.777 (0.939-5.518)	0.0543
Rectum	transcript	2.540 (1.941-4.245)	2.900 (2.233-4.866)	0.000481
	protein	2.434 (0.677-3.025)	2.947 (2.484-5.439)	0.00002
**Histologic grade**				
G1	transcript	2.121 (1.941-4.245)	3.061 (2.233-3.890)	0.438
	protein	2.398 (2.299-2.497)	2.030 (0.939-3.121)	0.99
G2	transcript	2.531 (2.008-4.245)	2.900 (2.005-4.875)	0.000004
	protein	2.415 (0.677-3.590)	3.137 (1.978-4.638)	0.000009
G3	transcript	2.716 (1.984-3.158)	2.994 (2.283-5.062)	0.0267
	protein	2.604 (1.229-3.021)	3.227 (2.501-5.518)	0.00124
**Dukes Scale**				
A	transcript	2.559 (2.008-3.308)	3.374 (2.794-4.866)	0.0374
	protein	2.454 (1.865-2.727)	2.796 (0.939-5.518)	0.149
B	transcript	2.569 (2.193-3.156)	3.077 (2.005-3.741)	0.00180
	protein	2.415 (1.394-2.988)	3.292 (2.841-4.309)	0.000069
C	transcript	2.662 (1.984-4.245)	2.898 (2.283-5.062)	0.00680
	protein	2.639 (0.677-3.590)	3.358 (2.484-5.439)	0.002
**Tumour stage**				
T1	transcript	3.108 (2.910-3.308)	3.247 (2.900-3.594)	0.99
	protein	2.410 (1.865-2.497)	2.951 (0.939-3.020)	0.517
T2	transcript	2.605 (2.338-3.158)	2.865 (2.761-3.665)	0.0181
	protein	2.727 (2.604-2.945)	3.665 (2.564-5.518)	0.175
T3	transcript	2.572 (1.984-4.245)	2.951 (2.005-5.662)	0.000221
	protein	2.464 (0.677-3.437)	3.212 (2.501-5.439)	0.000002
T4	transcript	2.706 (2.453-3.060)	3.161 (2.760-3.455)	0.0374
	protein	2.506 (1.883-3.590)	3.617 (2.484-4.413)	0.149

D.

FIH		primary cancerous tissues median (range)	histopathologically unchanged tissues median (range)	p value
	transcript	3.080 (2.576- 3.556)	3.131 (0.373-3.745)	0.583
	protein	2.965 (1.217-5.310)	2.785 (0.962-4.394)	0.0169
**Age (Years)**				
<60	transcript	3.154 (2.904-3.551)	3.265 (2.367-3.606)	0.885
	protein	2.989 (1.437-3.449)	2.889 (2.552-3.055)	0.248
>60	transcript	3.071 (2.576-3.556)	3.108 (0.373-3.745)	0.596
	protein	2.947 (1.217-5.310)	2.776 (0.963-4.394)	0.0257
**Gender**				
Female	transcript	3.161 (2.731-3.551)	3.233 (0.373-3.706)	0.411
	protein	2.927 (1.217-3.944)	2.803 (0.963-3.927)	0.267
Male	transcript	3.058 (2.576-3.556)	3.069 (2.367-3.745)	0.795
	protein	2.983 (1.949-5.310)	2.776 (1.066-4.394)	0.0210
**CRC localization**				
Proximal colon (cecum to transverse)	transcript	3.111 (2.756-3.556)	3.222 (2.692-3.705)	0.291
	protein	2.811 (1.217-3.843)	2.743 (0.963-4.207)	0.435
Distal colon (splenic flexture to sigmoid)	transcript	3.040 (2.576-3.500)	3.243 (2.414-3.745)	0.327
	protein	3.100 (1.949-5.310)	2.905 (1.066-4.394)	0.375
Rectum	transcript	3.128 (2.660-3.551)	3.063 (0.373-3.550)	0.117
	protein	3.036 (1.437-4.433)	2.798 (1.776-3.927)	0.031
**Histologic grade**				
G1	transcript	3.108 (3.055-3.161)	2.889 (2.845-2.933)	0.0869
	protein	3.059 (2.787-3.332)	2.665 (2.313-3.018)	0.99
G2	transcript	3.080 (2.576-3.511)	3.131 (1.768-3.706)	0.589
	protein	3.016 (1.217-5.310)	2.878 (0.963-4.394)	0.0226
G3	transcript	3.169 (2.773-3.551)	3.169 (0.373-3.745)	0.213
	protein	2.741 (2.276-2.741)	2.710 (2.139-3.452)	0.520
**Dukes Scale**				
A	transcript	3.041 (2.660-3.500)	2.883 (1.768-3.745)	0.631
	protein	3.033 (2.844-3.836)	2.742 (1.066-3.025)	0.0832
B	transcript	3.089 (2.576-3.403)	3.243 (2.692-3.523)	0.0522
	protein	2.916 (1.437-3.343)	2.745 (1.776-3.419)	0.213
C	transcript	3.154 (2.756-3.551)	3.169 (0.373-3.706)	0.570
	protein	3.011 (1.217-4.433)	2.817 (2.139-3.702)	0.153
**Tumour stage**				
T1	transcript	3.230 (3.061-3.399)	3.109 (2.744-3.474)	0.99
	protein	2.930 (2.844-3.016)	2.045 (1.066-3.025)	0.99
T2	transcript	3.206 (2.660-3.500)	3.169 (2.568-3.745)	0.749
	protein	3.022 (2.276-4.052)	2.675 (2.245-3.452)	0.465
T3	transcript	3.131 (2.731-3.551)	3.147 (0.373-3.606)	0.967
	protein	2.947 (1.437-3.836)	2.802 (1.776-3.702)	0.0544
T4	transcript	3.179 (2.576-3.511)	3.355 (3.244-3.700)	0.200
	protein	2.729 (1.217-4.443)	2.717 (2.411-2.954)	0.873

The PHD1/PHD2/PHD3/FIH transcript levels were standardized by the geometric mean of PBGD and hMRPL19 cDNA and expressed as decimal logarithm of multiples of these cDNA copies in calibrator. The amount of western blot-detected proteins was presented as the decimal logarithm PHD1/PHD2/PHD3/FIH to GAPDH band optical density ratio. p value - Unpaired, two-tailed t-test or U-Mann–Whitney test.
