# Supplementary figures and images for: Expression and DNA methylation levels of prolyl hydroxylases PHD1, PHD2, PHD3 and asparaginyl hydroxylase FIH in colorectal cancer
Source: BMC Cancer. 2013 Nov 6;13:526. doi: 10.1186/1471-2407-13-526 (PMC3828400; doi:10.1186/1471-2407-13-526)

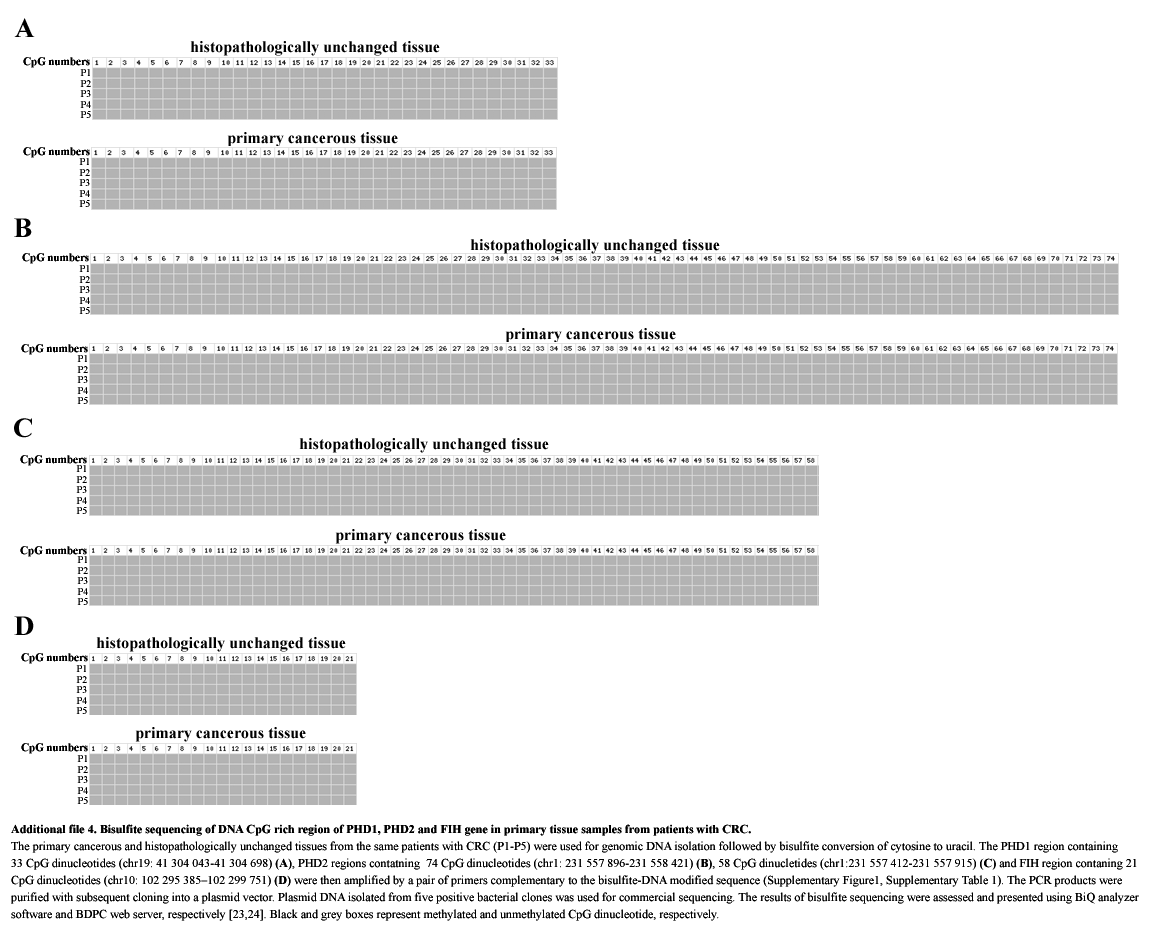

Supplement: Additional file 5 — Bisulfite sequencing of DNA CpG rich region of the PHD1, PHD2 and FIH genes in primary tissue samples from patients with CRC. [file 1471-2407-13-526-S5.tiff]
