# Supplementary material for: Expression and DNA methylation levels of prolyl hydroxylases PHD1, PHD2, PHD3 and asparaginyl hydroxylase FIH in colorectal cancer
Source: BMC Cancer. 2013 Nov 6;13:526. doi: 10.1186/1471-2407-13-526 (PMC3828400; doi:10.1186/1471-2407-13-526)
Supplement: Additional file 6 — DNA methylation assessment of the PHD1 , PHD2 and FIH gene regulatory region by HRM analysis. [file 1471-2407-13-526-S6.pdf]

A

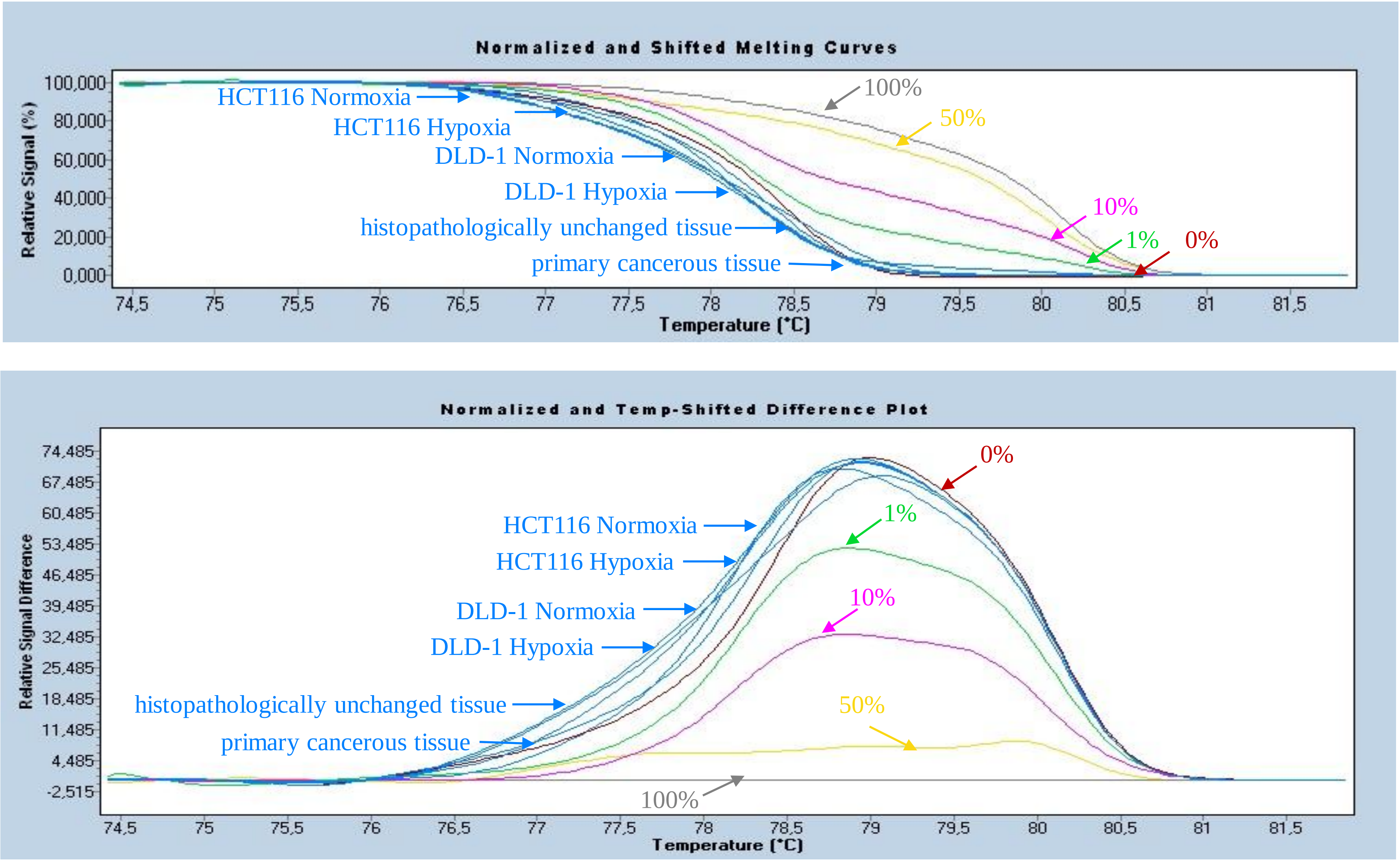
Normalized and Shifted Melting Curves
Relative Signal (%)
100,000
80,000
60,000
40,000
20,000
0,000
74,5
75
75,5
76
76,5
77
77,5
78
78,5
79
79,5
80
80,5
81
81,5
Temperature (°C)
HCT116 Normoxia
HCT116 Hypoxia
DLD-1 Normoxia
DLD-1 Hypoxia
histopathologically unchanged tissue
primary cancerous tissue
100%
50%
10%
1%
0%
Normalized and Temp-Shifted Difference Plot
Relative Signal Difference
74,485
67,485
60,485
53,485
46,485
39,485
32,485
25,485
18,485
11,485
4,485
-2,515
74,5
75
75,5
76
76,5
77
77,5
78
78,5
79
79,5
80
80,5
81
81,5
Temperature (°C)
0%
1%
HCT116 Normoxia
HCT116 Hypoxia
10%
DLD-1 Normoxia
DLD-1 Hypoxia
histopathologically unchanged tissue
50%
primary cancerous tissue
100%

B

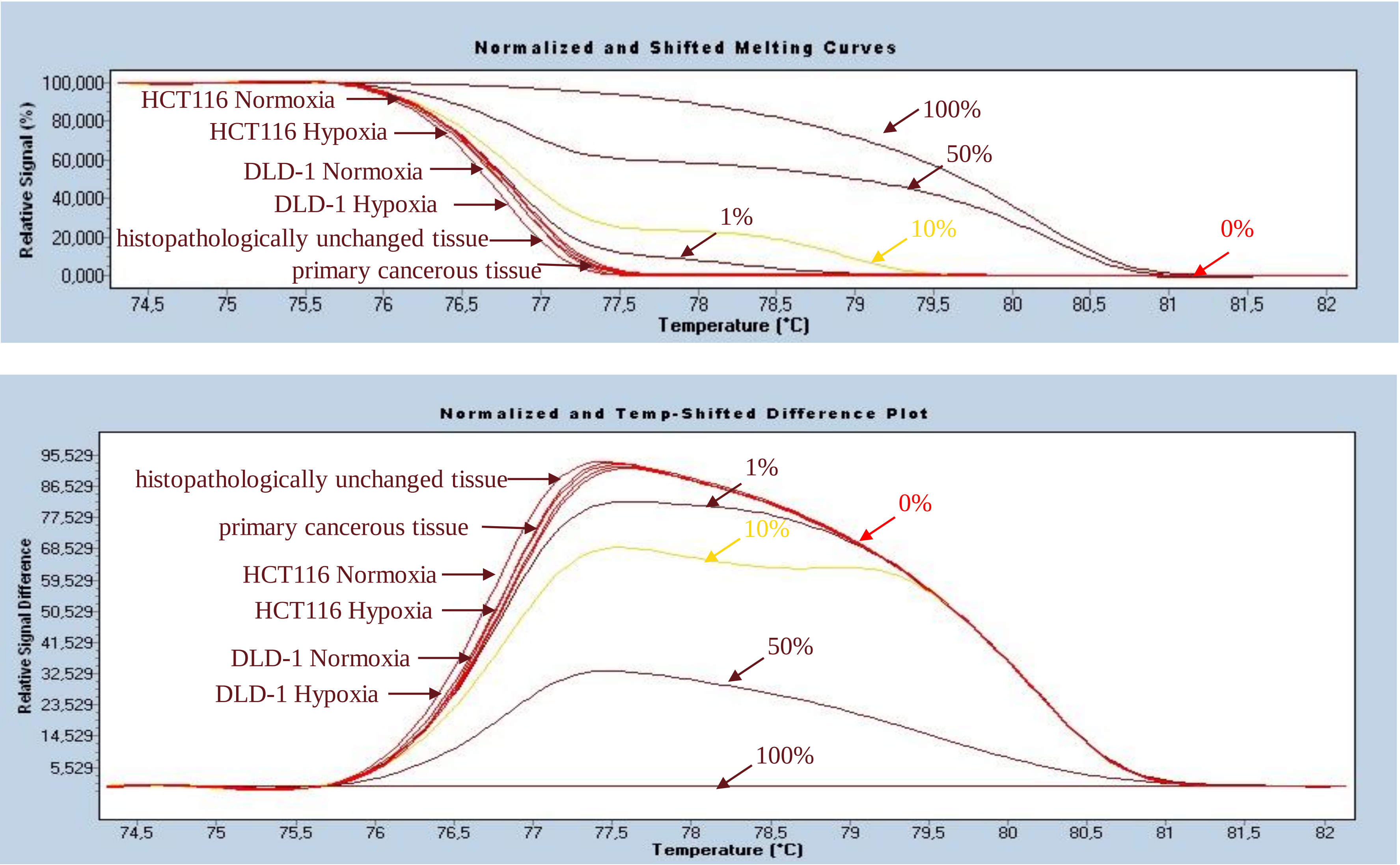

Normalized and Shifted Melting Curves
Relative Signal (%)
100,000
80,000
60,000
40,000
20,000
0,000
HCT116 Normoxia
HCT116 Hypoxia
DLD-1 Normoxia
DLD-1 Hypoxia
histopathologically unchanged tissue
primary cancerous tissue
100%
50%
1%
10%
0%
74,5
75
75,5
76
76,5
77
77,5
78
78,5
79
79,5
80
80,5
81
81,5
82
Temperature (°C)
Normalized and Temp-Shifted Difference Plot
Relative Signal Difference
95,529
86,529
77,529
68,529
59,529
50,529
41,529
32,529
23,529
14,529
5,529
histopathologically unchanged tissue
primary cancerous tissue
HCT116 Normoxia
HCT116 Hypoxia
DLD-1 Normoxia
DLD-1 Hypoxia
1%
0%
10%
50%
100%
74,5
75
75,5
76
76,5
77
77,5
78
78,5
79
79,5
80
80,5
81
81,5
82
Temperature (°C)

C

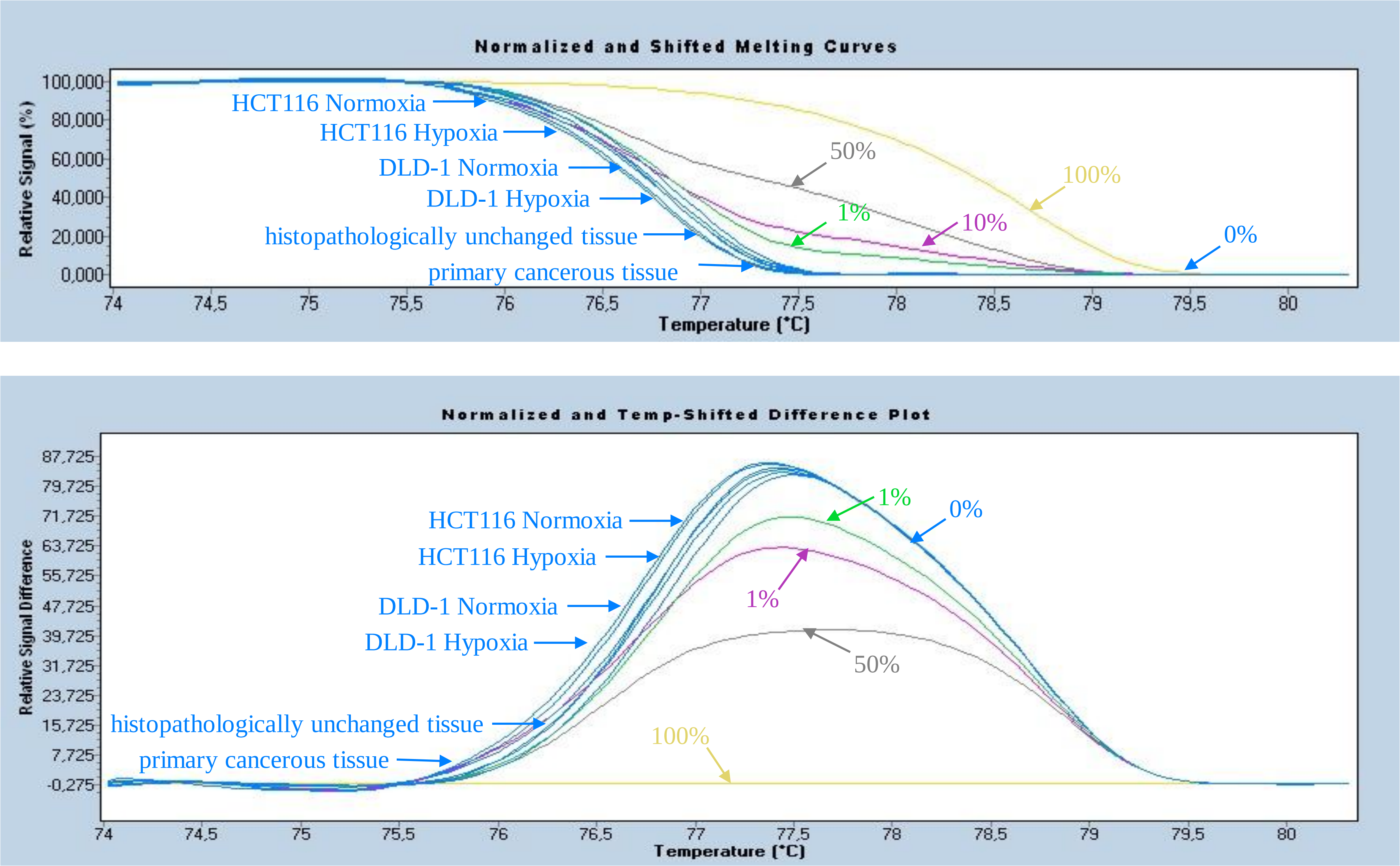

Normalized and Shifted Melting Curves
Relative Signal (%)
100,000
80,000
60,000
40,000
20,000
0,000
74
74,5
75
75,5
76
76,5
77
77,5
78
78,5
79
79,5
80
Temperature (°C)
HCT116 Normoxia
HCT116 Hypoxia
DLD-1 Normoxia
DLD-1 Hypoxia
histopathologically unchanged tissue
primary cancerous tissue
50%
100%
1%
10%
0%
Normalized and Temp-Shifted Difference Plot
Relative Signal Difference
87,725
79,725
71,725
63,725
55,725
47,725
39,725
31,725
23,725
15,725
7,725
-0,275
74
74,5
75
75,5
76
76,5
77
77,5
78
78,5
79
79,5
80
Temperature (°C)
HCT116 Normoxia
HCT116 Hypoxia
DLD-1 Normoxia
DLD-1 Hypoxia
histopathologically unchanged tissue
primary cancerous tissue
1%
0%
1%
50%
100%

D

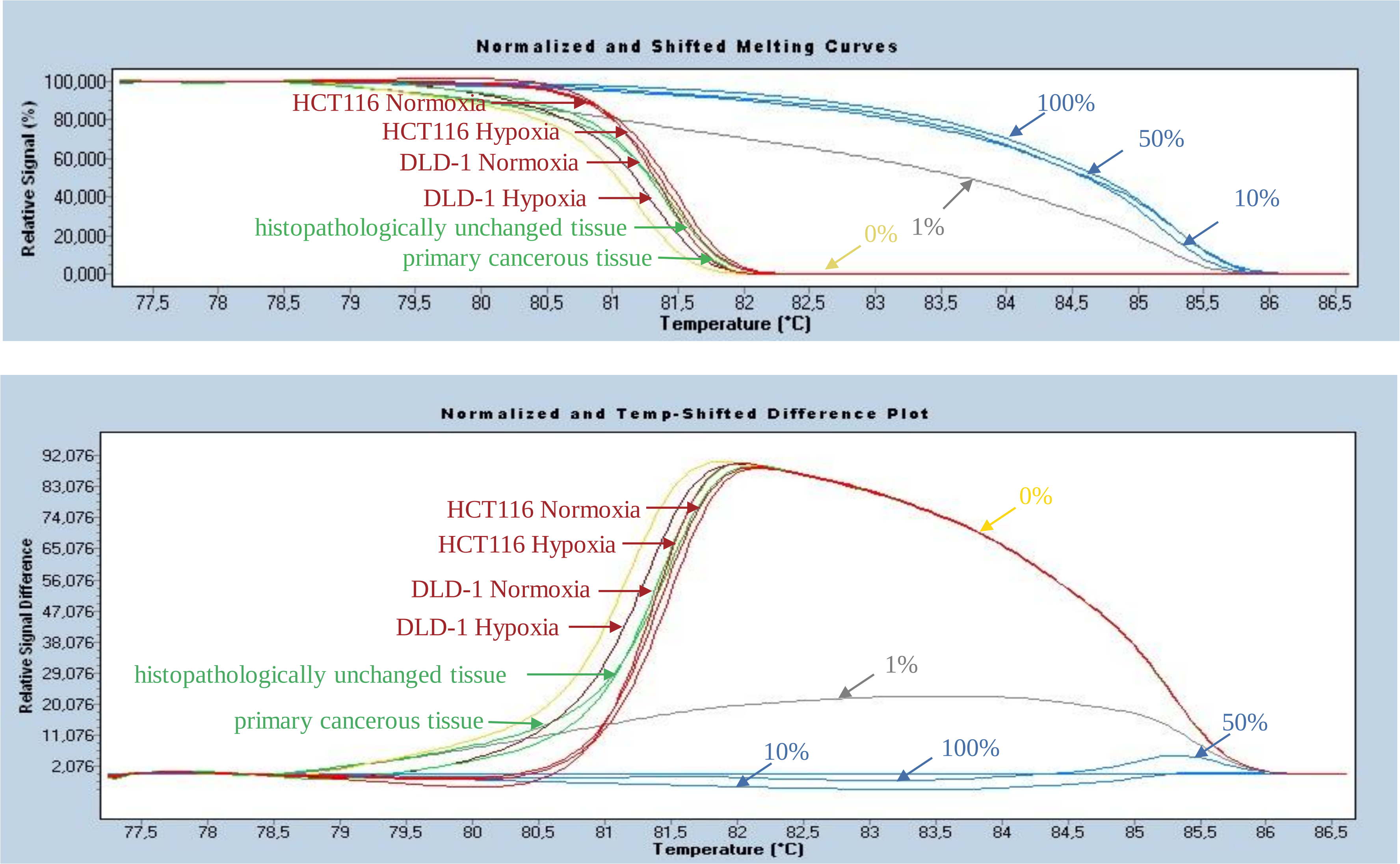
Normalized and Shifted Melting Curves
Relative Signal (%)
100,000
80,000
60,000
40,000
20,000
0,000
77,5
78
78,5
79
79,5
80
80,5
81
81,5
82
82,5
83
83,5
84
84,5
85
85,5
86
86,5
Temperature (°C)
HCT116 Normoxia
HCT116 Hypoxia
DLD-1 Normoxia
DLD-1 Hypoxia
histopathologically unchanged tissue
primary cancerous tissue
100%
50%
10%
1%
0%
Normalized and Temp-Shifted Difference Plot
Relative Signal Difference
92,076
83,076
74,076
65,076
56,076
47,076
38,076
29,076
20,076
11,076
2,076
77,5
78
78,5
79
79,5
80
80,5
81
81,5
82
82,5
83
83,5
84
84,5
85
85,5
86
86,5
Temperature (°C)
HCT116 Normoxia
HCT116 Hypoxia
DLD-1 Normoxia
DLD-1 Hypoxia
histopathologically unchanged tissue
primary cancerous tissue
0%
1%
50%
10%
100%

E

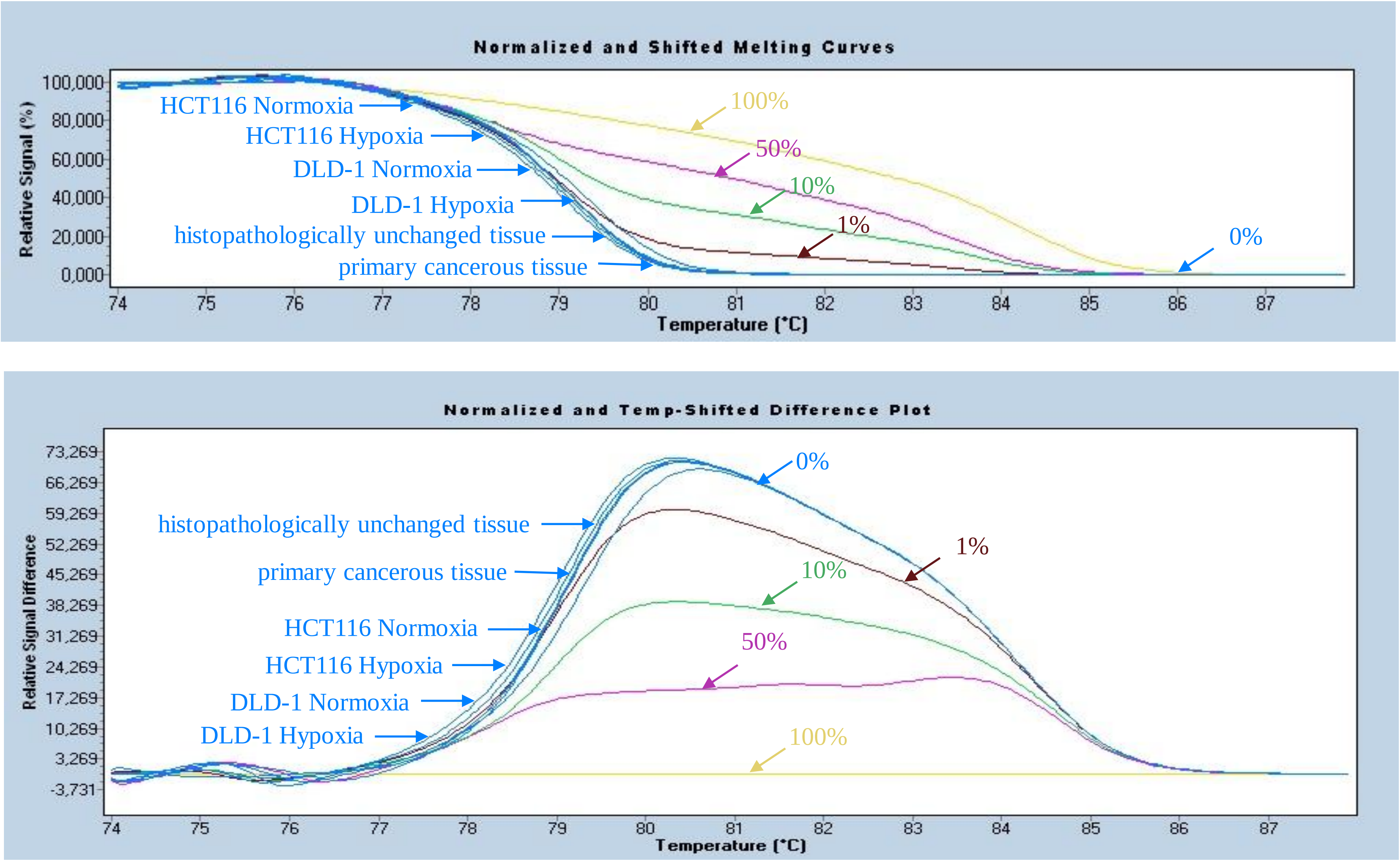

Normalized and Shifted Melting Curves
Relative Signal (%)
100,000
80,000
60,000
40,000
20,000
0,000
74
75
76
77
78
79
80
81
82
83
84
85
86
87
Temperature (°C)
HCT116 Normoxia
HCT116 Hypoxia
DLD-1 Normoxia
DLD-1 Hypoxia
histopathologically unchanged tissue
primary cancerous tissue
100%
50%
10%
1%
0%
Normalized and Temp-Shifted Difference Plot
Relative Signal Difference
73,269
66,269
59,269
52,269
45,269
38,269
31,269
24,269
17,269
10,269
3,269
-3,731
74
75
76
77
78
79
80
81
82
83
84
85
86
87
Temperature (°C)
histopathologically unchanged tissue
primary cancerous tissue
HCT116 Normoxia
HCT116 Hypoxia
DLD-1 Normoxia
DLD-1 Hypoxia
0%
1%
10%
50%
100%

F

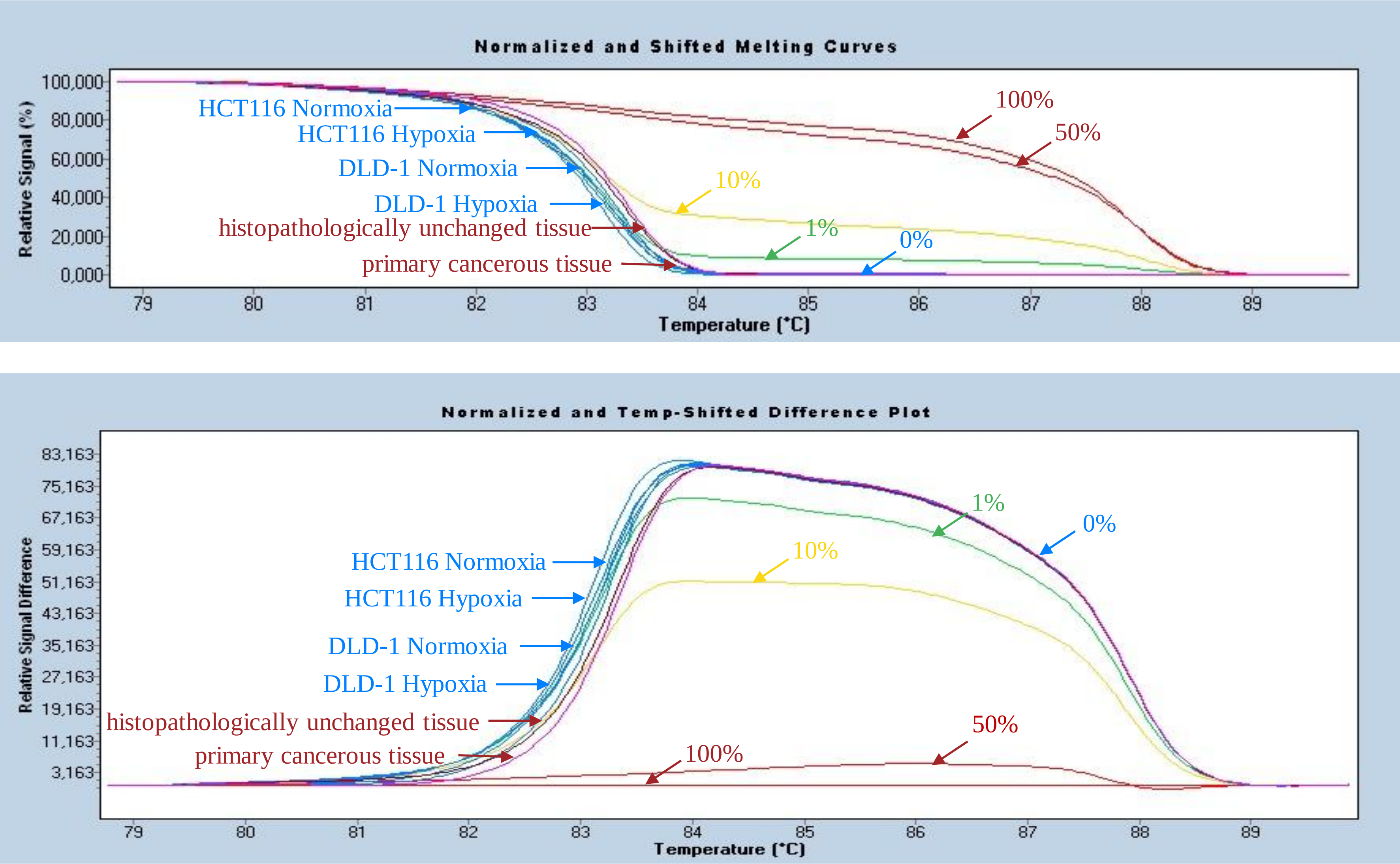
Normalized and Shifted Melting Curves
Relative Signal (%)
100,000
80,000
60,000
40,000
20,000
0,000
79
80
81
82
83
84
85
86
87
88
89
Temperature (°C)
HCT116 Normoxia
HCT116 Hypoxia
DLD-1 Normoxia
DLD-1 Hypoxia
histopathologically unchanged tissue
primary cancerous tissue
100%
50%
10%
1%
0%
Normalized and Temp-Shifted Difference Plot
Relative Signal Difference
83,163
75,163
67,163
59,163
51,163
43,163
35,163
27,163
19,163
11,163
3,163
79
80
81
82
83
84
85
86
87
88
89
Temperature (°C)
HCT116 Normoxia
HCT116 Hypoxia
DLD-1 Normoxia
DLD-1 Hypoxia
histopathologically unchanged tissue
primary cancerous tissue
1%
0%
10%
50%
100%

Additional file 5. DNA methylation assessment of *PHD1, PHD2* and *FIH* gene regulatory region by HRM analysis.
Methylation percentage of three DNA fragments within the *PHD1*, *PHD2* and *FIH* CpG island (Additional file 1, Additional file 2) was determined by Real Time PCR amplification of bisulfite treated standard, patient DNA and cell line DNA followed by comparison of their HRM profiles. DNA standards were prepared by mixing different ratios of methylated and non-methylated bisulfite treated DNA. HRM methylation analysis was performed using Light Cycler®480 Gene Scanning software, Roche Diagnostics GmbH (Mannheim, Germany). Each PCR amplification and HRM profile analysis was performed in triplicate. A, B, C, D, E and F represent HRM profiles of standard, patient and cell line DNA PCR product for primers numbered PHD1.1, PHD1.2, PHD2.1, PHD2.2, PHD2.3 and FIH.1 respectively.
